# Characterization of hemocytes and hematopoietic cells of a freshwater crayfish based on single-cell transcriptome analysis

**DOI:** 10.1016/j.isci.2022.104850

**Published:** 2022-08-02

**Authors:** Irene Söderhäll, Erik Fasterius, Charlotta Ekblom, Kenneth Söderhäll

**Affiliations:** 1 Department of Organismal Biology, Uppsala University, Norbyvägen 18 A, SE752 36 Uppsala, Sweden; 2 National Bioinformatics Infrastructure Sweden, Science for Life Laboratory, Stockholm University, Tomtebodavägen 23, SE171 65 Solna, Sweden

**Keywords:** Zoology, Omics, Transcriptomics

## Abstract

Crustaceans constitute a species-rich and ecologically important animal group, and their circulating blood cells (hemocytes) are of critical importance in immunity as key players in pathogen recognition, phagocytosis, melanization, and antimicrobial defense. To gain a better understanding of the immune responses to different pathogens, it is crucial that we identify different hemocyte subpopulations with different functions and gain a better understanding of how these cells are formed. Here, we performed single-cell RNA sequencing of isolated hematopoietic tissue (HPT) cells and hemocytes from the crayfish *Pacifastacus leniusculus* to identify hitherto undescribed hemocyte types in the circulation and show that the circulating cells are more diversified than previously recognized. In addition, we discovered cell populations in the HPT with clear precursor characteristics as well as cells involved in iron homeostasis, representing a previously undiscovered cell type. These findings may improve our understanding of hematopoietic stem cell regulation in crustaceans and other animals.

## Highlights

• Single-cell RNA sequencing of hematopoietic cell types reveals new cell types
• One cell type contains iron homeostasis-associated transcripts
• Hemocytes and hematopoietic cells differ in their transcript profiles
• Prophenoloxidase is only expressed in hemocytes

## Graphical abstract

**Figure:**
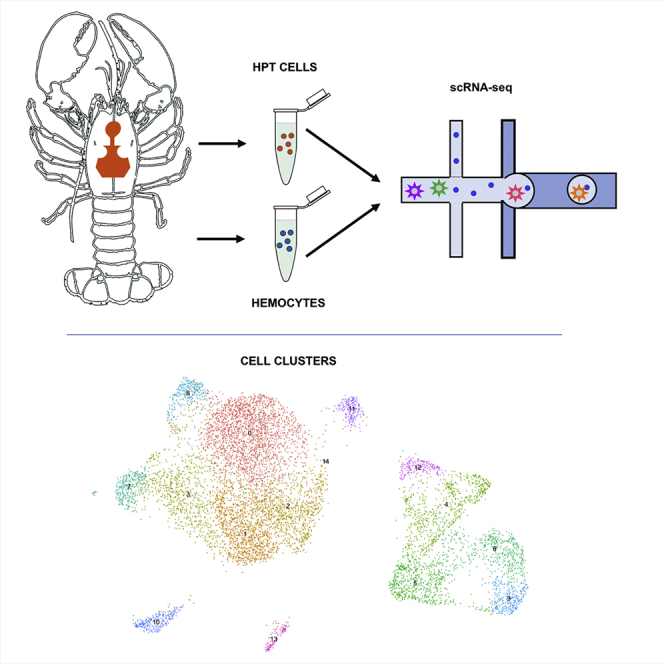

## Introduction

Seas, lakes, and streams are habitats for one of the largest groups of animals on our planet, namely crustaceans, in addition to an immense number of microorganisms. In the Baltic Sea alone and its inflows from north to south, a research group has identified more than 6.8 million genes from various microorganisms (Alneberg et al., 2018). Living in an ocean or lake environment therefore means being surrounded by large numbers of different microorganisms, as well as potential pathogens. For crustaceans that lack the adaptive immune system found in vertebrates, it is therefore absolutely crucial for their survival that they have an active native innate defense. The blood circulation of crustaceans is therefore filled with immune cells so-called hemocytes, which perform various tasks for the defense of the animals, including healing injuries, preventing pathogens through phagocytosis and encapsulation, and secreting antimicrobial substances that kill or inhibit the growth of pathogens (Söderhäll, 2016). The function of hemocytes has been studied in these animals for many decades, and several different immune pathways have been mapped in detail, such as the so-called proPO-activating system (Cerenius and Söderhäll, 2021). This reaction is responsible for the melanization process that produces toxic intermediates and helps to encapsulate larger pathogens in melanin layers (Cerenius et al., 2008). In addition, during activation of the proPO-activating system process, a number of different active peptides are produced, with both antimicrobial and hemocyte synthesis stimulating effects (Jearaphunt et al., 2014; Lee and Söderhäll, 2001; Sirikharin et al., 2020; Wang et al., 2001). Hemocytes are also important players in wound healing (Cerenius et al., 2010; Vogt, 2022). The hematopoietic tissue of crayfish harbors multipotent precursor cells with immune functions, but also cells which serve as the first generation of precursor cells for adult neurogenesis in the olfactory lobes of the brain (Benton et al., 2014).

Hemocytes in crayfish have traditionally been divided into groups according to morphological characteristics, including the amount and type of granules in the cytoplasm (Johansson et al., 2000; Söderhäll, 2016). Cells were then named according to these criteria as hyaline (HC, without granules), semigranular (SGC, with few and less electron-dense granules), and granular hemocytes (GC, filled with large electron-dense granules). It is possible to separate these cell types by means of gradient centrifugation and thereby be able to study the function of each of these types (Söderhäll and Smith, 1983). In recent years, several methods have been used to characterize these groups, such as monoclonal antibodies (Lin et al., 2007; van de Braak et al., 2000; Xing et al., 2017), flow cytometry (Sequeira et al., 1996; Zhou et al., 2018), and proteomic analysis to find specific markers for these groups (Söderhäll and Junkunlo, 2019; Wu et al., 2008). These studies have all been based on the traditional division into HC, SGC, and GC. However, several studies of immunological functions indicate that this division is very rough and that many more categories of hemocytes with specific functions likely exist. *In situ* hybridization analysis of mRNA expression in hemocytes (and also in their hematopoietic precursors) reveals that several genes are only expressed in a small subset of hemocytes. This notion applies, for example, to the PDGF/VEGF receptor family protein and transglutaminase (Junkunlo et al., 2017, 2020).

Similarly, hemocytes have also been classified according to morphological characteristics in insects. However, recently, several researchers have used single-cell RNA sequencing and thus have been able to identify specific subgroups of hemocytes in two of the most studied insect species, *Drosophila melanogaster* (Cho et al., 2020) and *Anopheles gambiae* (Raddi et al., 2020). These studies have to some extent confirmed the relevance of the previously morphologically based division, but several interesting subgroups have been identified, indicating a more specific immune system than previously realized. In the fruit fly Drosophila, single-cell analysis of the hemocyte-producing larval lymph gland with trajectory analysis suggests a more detailed differentiation process than previously known (Cho et al., 2020). How insect hemocytes are formed and how this process is regulated has been studied in detail in Drosophila, which often serves as a model animal for invertebrates in general and arthropods in particular (Banerjee et al., 2019; Morin-Poulard et al., 2021). Given its well-mapped genetics and short generation time, studies of the fruit fly have contributed to several important discoveries of basic principles in molecular, cell, and developmental biology. However, it is important to understand that although Drosophila offers many benefits for different studies, it represents only one organism within the enormous diversity of arthropods and invertebrates. The fruit fly has a very short lifetime, and its need for an immune defense most likely differs extensively from that of large and long-lived crustaceans, which are also highly prone to mechanical damage in their natural habitats. In addition to insects, there are many more arthropod groups, such as spiders, mites, ticks, scorpions, horseshoe crabs, centipedes, and crustaceans. The last group is a highly diversified group with between 40,000 and 60,000 species (Vogt, 2022). To enable broader comparative studies of the development of arthropods and their immune systems and function, it is thus necessary to investigate and develop other model systems. Regarding immunity, the commercially important decapod crustaceans have thus far dominated this type of research (Vogt, 2022). Although there are many similarities, especially basic cell signaling pathways and regulatory transcription factors, important differences exist between the immune systems of, for example, the crayfish and the fruit fly. These organisms have different types of antimicrobial substances, different growth factors, and markedly different cell types. Hemocyte formation is an important process for the immune system, as a large proportion of the hemocytes are consumed after injury and infection (Söderhäll, 2016; Söderhäll et al., 2003). In crayfish, hematopoiesis is an ongoing process throughout the life of the animal. However, in Drosophila, hematopoiesis mainly occurs during the embryonic and larval stages.

When a crayfish suffers from an infection, a significant portion of hemocytes are consumed and their numbers in the circulation decrease but rapidly return to a normal level (Persson et al., 1987). This process is accomplished by the release of hemocytes from the hematopoietic tissue (HPT) (Hammond and Smith, 2002; Söderhäll et al., 2003). Studies of mRNA expression in hemocytes before and after an infection often show large differences in certain transcripts. What makes such studies very difficult to interpret is that information about the composition of different hemocyte types in the circulation is rarely given, and whether the specific response results from the induction of new synthesis and/or release of a specific population of hemocytes remains unknown (Ekblom et al., 2021). A similar question is whether specific cell types are released from the HPT in response to the induction of adult neurogenesis (Beltz and Benton, 2017), or injury. Cells released from the HPT have been shown to not only be attracted to the neurogenic niche (Benton et al., 2011) but also to divide in the niche and migrate in a stream to the proliferating clusters in the olfactory lobe of the brain (Benton et al., 2014, 2022). To obtain more information about possible neuronal or glial precursor cells, as well as specific subpopulations of hemocytes and their precursors, we have here performed single-cell RNA sequencing of isolated HPT cells and hemocytes from a crustacean, the crayfish *Pacifastacus leniusculus.*

## Results and discussion

### Transcriptomic profiling of single HPT cells and hemocytes

**Figure 1:**
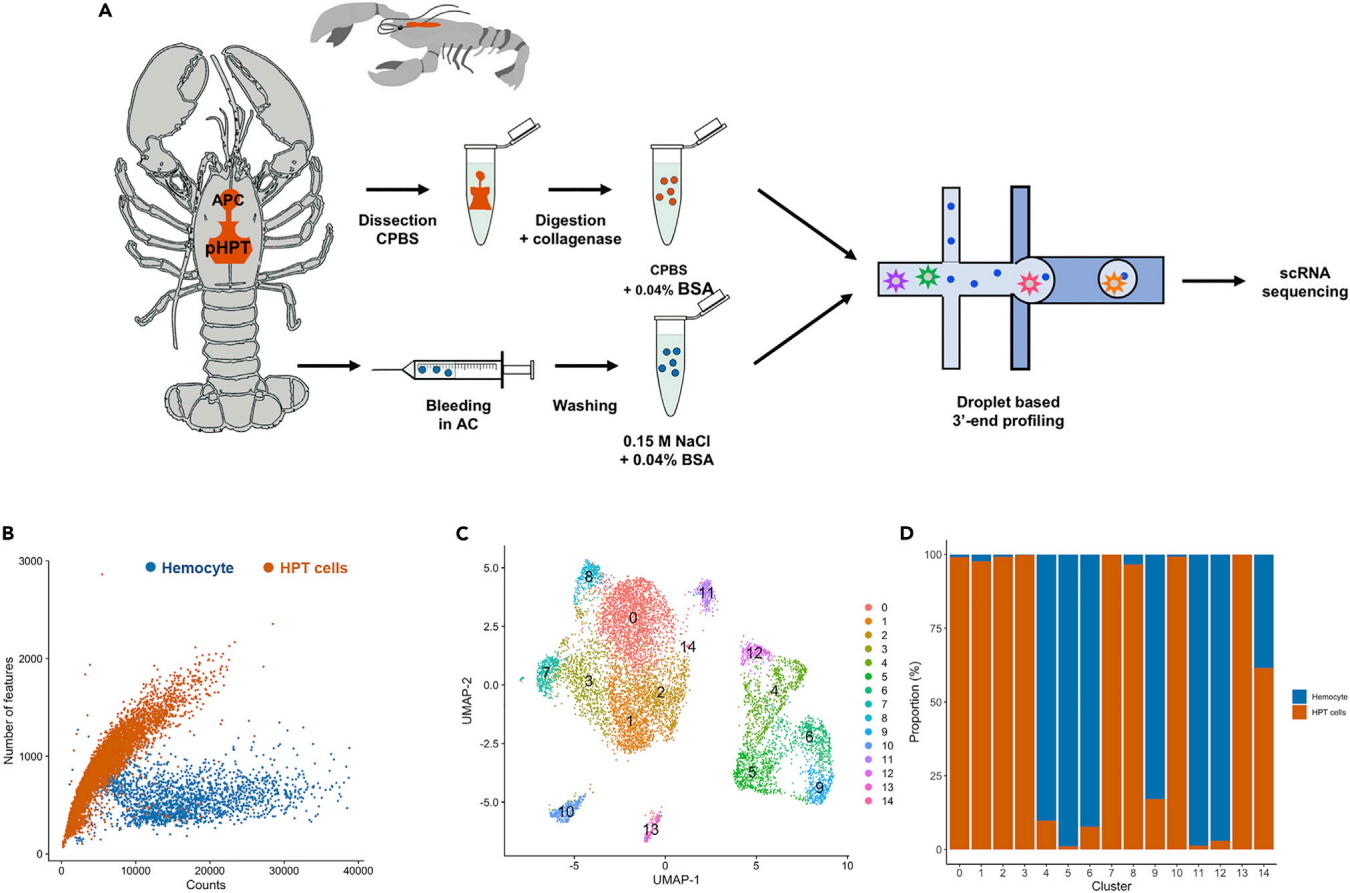
Clustering of different cell types in *P. leniusculus* circulation and HPT, respectively, identified by single-cell RNA sequencing (A) Workflow of sample preparation for droplet-based scRNA sequencing. (B) Number of features detected per cell (Y axis) versus number of counts per feature (x axis) in HPT cells (red) and hemocytes (blue), respectively. (C) UMAP plot after quality correction and integration of HPT cell and hemocyte samples. (D) Sample (HPT cells or hemocytes) distribution in each cluster as proportion of all cells in the specific cluster.

To uncover information on crayfish hemocyte variability, the functional diversity of different crayfish hemocyte types, and the origins of these cells, we applied single-cell RNA-sequence analysis to hemocytes from naive crayfish and cells from dissociated hematopoietic tissues (HPTs) including the anterior proliferation center (APC) (Noonin et al., 2012), using 10x Genomics droplet-based sequencing. Two independent sequencing libraries were prepared from 10,000 hemocytes and 10,000 dissociated HPT cells (Figure 1A and S1). Following library preparation and sequencing the transcripts were mapped to the *P. leniusculus* transcriptome ( GenBank :PRJNA259594). Given that a complete genome is not available for this species, sequences known to cover the same transcript were merged and then named “features” in the following text.

Quality controls included removal of cells with fewer than 10 features and cells with more than 40,000 Unique Molecular Identifiers (UMIs), cells with greater than 30% mitochondrial genes, and greater than 7.5% ribosomal genes. Moreover, features present in fewer than 3 cells were removed, as were cells with more than 3000 features in total. The data from the two libraries were then integrated using Seurat (see Methods).

A total number of 6585 HPT cells and 2546 hemocytes were retained for further detailed profiling. The two populations differed substantially in library complexity. The HPT cells had a median of 4460 UMIs and 830 features per cell, whereas the hemocyte population showed transcriptomes of lesser complexity with more counts covering a lower number of features per cell (12995 and 619, respectively; Figure 1B and S2). Based on these results, it is clear that hemocytes are more specialized cells expressing few genes at high levels, compared to the less differentiated precursor cells in the HPT.

### Single-cell transcriptome exploration without an annotated genome

Given that the entire *P. leniusculus* genome is not sequenced and annotated and we instead used transcriptome data, it was not possible to fully identify all transcripts in our scRNA analysis. A large proportion of the transcripts also appeared to be lncRNAs or transposons. These sequences are known to be exceptionally frequent in decapod crustaceans with large genomes (Alfsnes et al., 2017; Uengwetwanit et al., 2021). In addition, the genome size of *P. leniusculus* has been estimated to be approximately 18 Gb in an ongoing sequencing project. Recently, the genomes of two shrimp species, *Fenneropenaeus chinensis* and *Litopenaeus vannamei*, were found to contain 19.12% and 16.17% transposable elements (TEs), respectively (Yuan et al., 2021). Due to the lack of an annotated genome of *P. leniusculus*, this single-cell sequencing study was not exclusively focused on gene expression in contrast to most scRNA studies. Our study, instead, revealed very high expression of several TEs, such as retrovirus-related pol proteins, gag, and jockey-like TEs (Finnegan, 1997; Mizrokhi and Mazo, 1990). Notably, the expression of such TEs was considerably increased in HPT cells compared with mature hemocytes, which may reflect the “selfish” characteristic of TEs exhibiting expression in highly proliferating cells. How crayfish can cope with such high expression levels of TEs and whether they can use some of these TEs for their own benefit as regulatory elements is an interesting question (Goodier, 2016), but outside the aim of this study. Thus, to obtain as much information as possible from the scRNA-sequencing experiment, and to be able to identify different cell types and possible markers, we concentrated our analysis on the transcripts encoding known proteins. Transcripts are named by their contig name in the following text.

### Characterization of major hemocyte and HPT cell clusters

Clustering of the integrated single-cell transcriptomes clearly showed that the sample origin (hemocytes or HPT cells) was the main driving factor behind clustering (Figure S2A). The integrated transcriptome data revealed 14 major cell clusters (Figure 1C), where the majority of hemocytes clustered close together in a separate cloud (right in Figures 1C and S2A). In addition, the majority of the HPT cells clustered in another distinct cloud, together with two minor isolated clusters (CL10 and CL13) (left in Figures 1C and S2A). However, some of the individual clusters contained cells from both samples although each cluster was clearly dominated by either HPT cells or hemocytes (Figures 1D and S2A–S2C). The mixture of cells observed in some clusters may partly be attributed to a few circulating hemocytes that are tightly attached to the hematopoietic tissue, and are not removed during tissue dissection and washing (Figures S1A–S1C). Such “contaminating” hemocytes do however never represent more than 1% of the total cell population.

**Figure 2:**
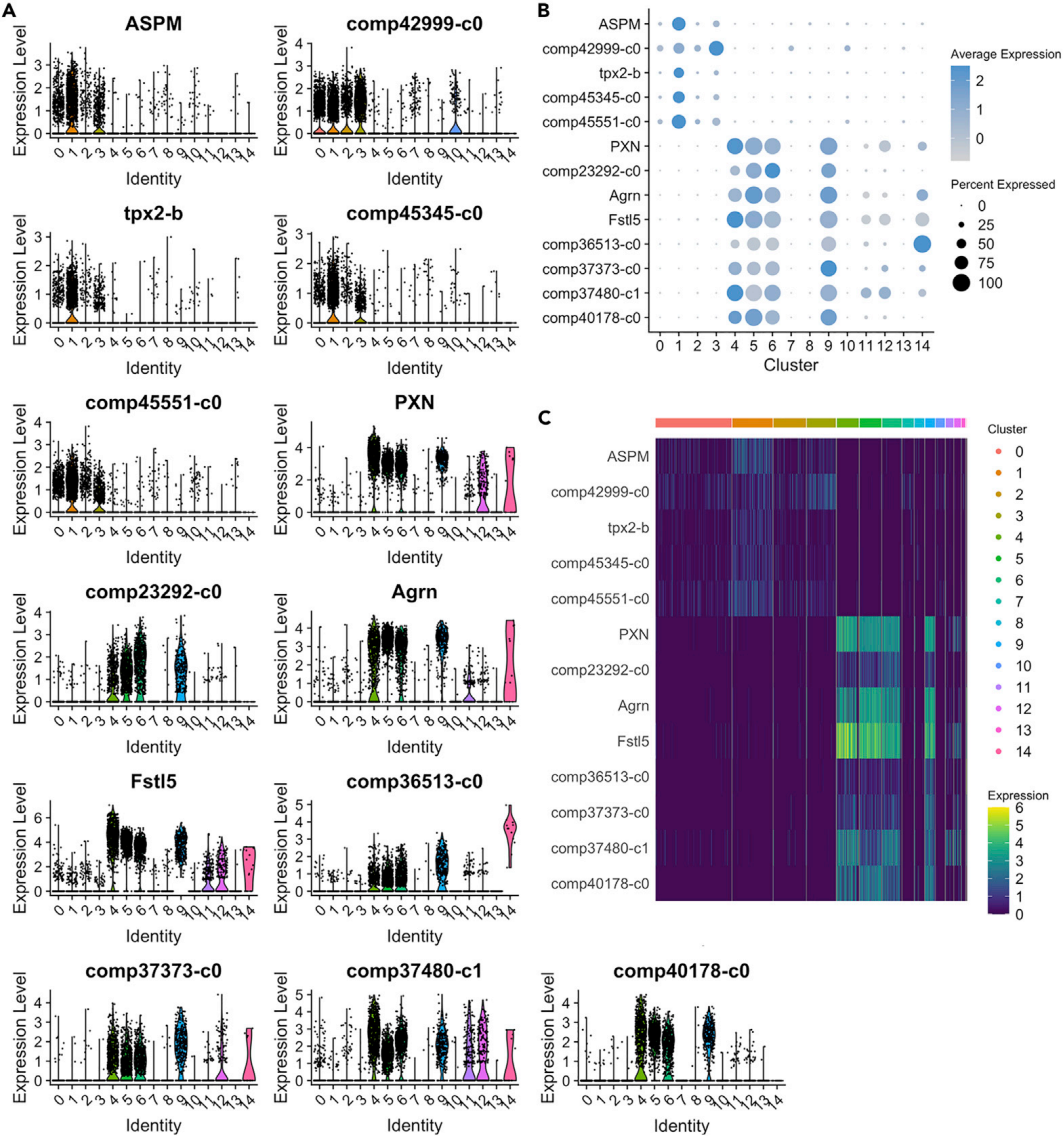
Major transcripts discriminating circulating hemocytes and HPT cells (A) Violin plots showing expression levels and cluster distribution for five selected markers for HPT cells, and eight selected markers for hemocytes. (B) Dot plots and (C) Heatmap for these markers.

A few hemocyte-sample cells are also detected in the left cloud in CL0-CL3, CL7, CL10, and CL14 and these cells are most likely immature precursor hemocytes which are present in the circulation (Figures 1D and S2C). Notable is that one hemocyte cluster (CL11) is closely connected to the left cloud, which is dominated by HPT cells. This suggests that cells in the CL11 are less differentiated hemocytes or even circulating prohemocytes (Figures 1C and 1D). The cluster in the left cloud is dominated by the large CL0-CL3, which contain by far the highest number of cells (Figure S1C) and these four clusters had characteristics that strongly suggest that they are proliferating cells through the expression of a number of genes linked to the mitotic process. A selection of these are illustrated in the different plots in Figures 2A and 2B, which also show that these transcripts are hardly detectable in the circulating hemocytes (Figures 2A–2C and S3A).

The precursor cell markers in HPT include ASPM (abnormal spindle-like microcephaly associated protein). In human cells, ASPM accumulates at the onset of mitosis and localizes to the spindle pole, where it regulates orientation of the mitotic spindle apparatus (Gai et al., 2016; Safieddine et al., 2021). Other mitosis-associated transcripts characteristic of the left cloud showed similarity to a putative protein MIS12 homolog (comp45551_c0), a protein regulator of cytokinesis 1-like GBEV01006525.1 (comp45345_c0), a nucleoporin NUP43 (comp42999_c0), and Tpx2-B (Figures 2A, 2B, and S3A).

In the right cloud, including CL4-CL6, CL9, CL11, and CL12, several immune response-related transcripts were identified as clearly separated from HPT cells; prophenoloxidase (proPO,comp37373_c0, GenBank:X83494.1); two Kazal domain protease inhibitors, Agrn and Fstl5; peroxinectin (PXN, GenBank:X91409.1); comp37480_c0, a transcript encoding an LPMO_10 domain putative chitin binding protein; and astacidin2 (comp36513_c0, Genbank:DQ822206) (Figures 2A, 2B, and S3A). Expression of proPO was detected in the majority of hemocyte clusters (right cloud) with some differences in the proportion of proPO-expressing cells noted in the separate clusters (Figure 2A). However, less than 0.5% of hematopoietic cells showed proPO expression (Figures 2A and 2B). This result confirms our previous findings indicating that mRNA proPO expression is induced after release from the HPT when hemocytes enter the circulation (Söderhäll et al., 2003).

Crustin antimicrobial peptides (Smith et al., 2008) are usually expressed at very high levels in crustaceans. In this scRNA analysis, we detected six different crustin transcripts (Figure S3B), all of which were highly expressed in hemocyte clusters. Two crustin transcripts were also present in HPT (Figure S3B). The finding of crustin 3 (comp40178_c0) and a previously uncharacterized crustin-23292 as specific for mature hemocytes is also consistent with our previous findings about the expression pattern of crustin 1 (comp36491_c0), crustin 2 (comp36467_c0), and crustin 3 mRNA in HPT cells and hemocytes (Jiravanichpaisal et al., 2007b). When hemocytes are separated by Percoll gradient centrifugation into granular cell (GC) and semigranular cell (SGC) fractions, crustin 3 protein is specifically expressed in the GC fraction (Söderhäll and Junkunlo, 2019). According to the fractions presented in the violin plots (Figure 2A), GC may be dominant in CL5 and CL9, but is also frequent in CL4 and CL6. Nonetheless, it is clear that not all hemocytes express this specific antimicrobial peptide; thus, different hemocyte subsets clearly have different functions. Accordingly, CL11 and CL12 show a very low proportion of cells expressing crustin 3, indicating that these clusters are dominated by the SGC morphotype. We analyzed the proteome data from our earlier study (Söderhäll and Junkunlo, 2019) with respect to the protein expression of crustin 1,2, and 3 in GC, SGC, and total HPT cells, and the expression pattern is clearly consistent with the new scRNA-sequencing data showing high expression of crustin 1–2 in all hemocytes. Crustin 1–2 are also expressed in HPT. In contrast, crustin 3 is specific for the GC fraction and absent from the HPT (Figure S3C) (Söderhäll and Junkunlo, 2019).

### Heterogeneity of HPT precursor cells

**Figure 3:**
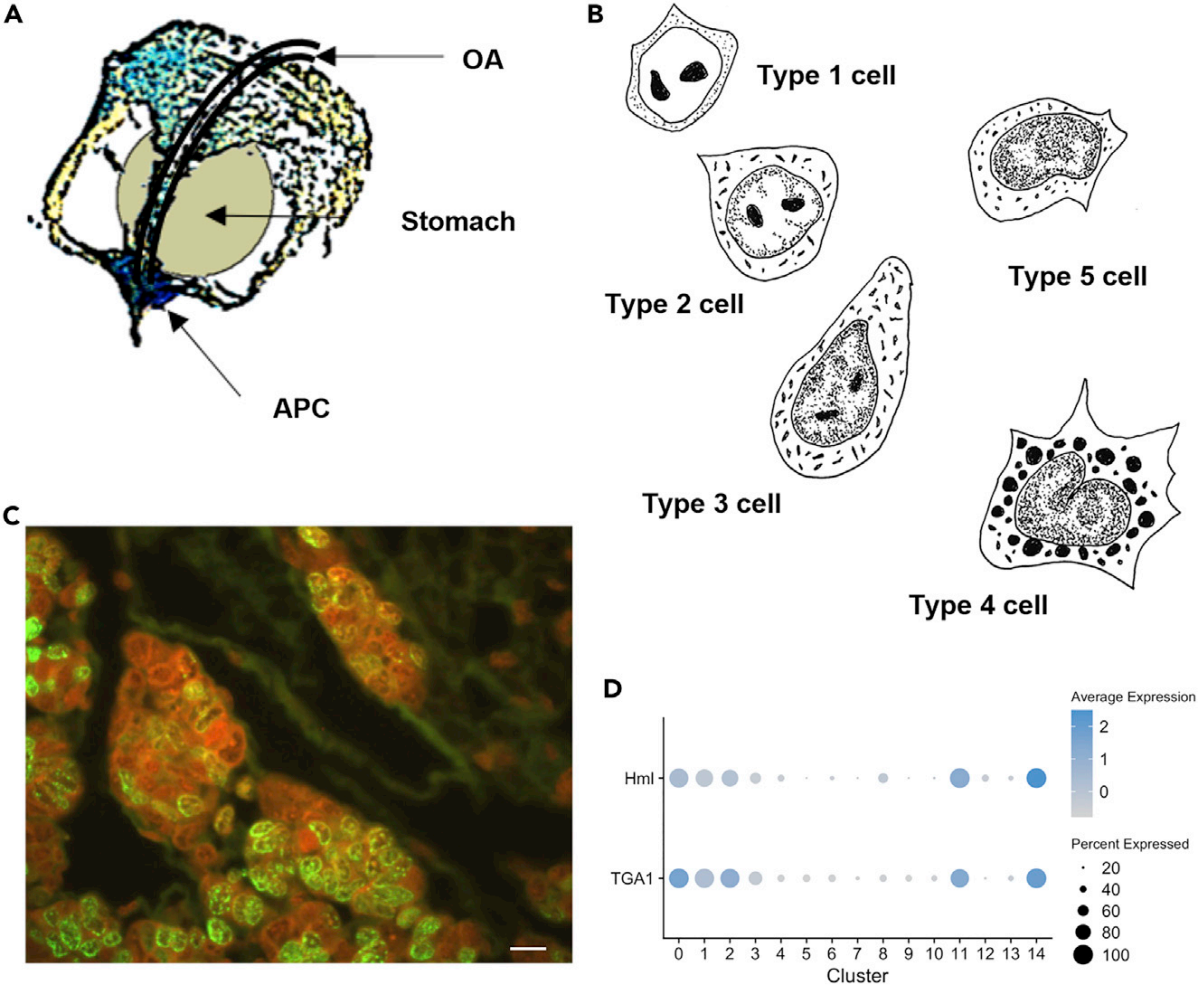
Characterization and localization of the *P. leniusculus* HPT (A) Illustration showing the localization of the HPT underneath the carapace and covering the stomach in the anterior thorax of crayfish, APC is the anterior posterior center (Noonin et al., 2012), and OA represents the ophthalmic artery. (B) Cell types within the HPT illustrated after Chaga et al. (1995). (C) BrdU (FITC green) incorporation in the HPT lobules 6 h after injection as previously described (Söderhäll et al., 2003). The bar indicates 15 μm in length. (D) Dotplot showing expression and cluster distribution of hemolectin (Hml) and transglutaminase 1 (TGA1).

We then analyzed the clustering of HPT cells (including APC) in the left cloud to characterize the major precursor cell types and putative differentiation pathways. The crayfish HPT differs from the lymph gland of Drosophila in that mature hemocytes are not part of the tissue. In contrast, the Drosophila lymph gland cortical zone contains mature plasmatocytes, proPO-expressing crystal cells, and occasionally lamellocytes (Jung et al., 2005). In our earlier studies of the HPT, we were not able to identify any distinct zones similar to the medullary and cortical zone of the Drosophila lymph gland, except for the crayfish APC in the core frontal region, where highly proliferating cells reside (Benton et al., 2022; Noonin et al., 2012). The HPT is a very thin sheet and contains cells packed in small lobules surrounded by extracellular matrix proteins, and the whole tissue is tightly attached underneath the carapace, covers the stomach, and extends toward the core frontale, where the APC (Noonin et al., 2012) is located (Figure 3A). The first characterization of *P. leniusculus* hematopoietic tissue made by Chaga et al. (1995) identified five different cell types differing in ultrastructural morphology based on TEM (schematic drawings in Figure 3B). The cell type considered to be less differentiated was localized to the apical tip of the lobules in which all cells are tightly packed (named as type 1), while the majority of dividing cells were scattered throughout the tissue. This result was also confirmed by BrdU labeling, as shown in Figure 3C.

In this integrated scRNA sequence analysis of both hemocytes and HPT cells, we identified nine distinct cell clusters with cells from the HPT sample, two of which were separated from the main cloud (CL10 and CL13), which will be discussed later below. One of the clusters (CL14) contained only 12 cells; thus, it will not be further analyzed here.

**Figure 4:**
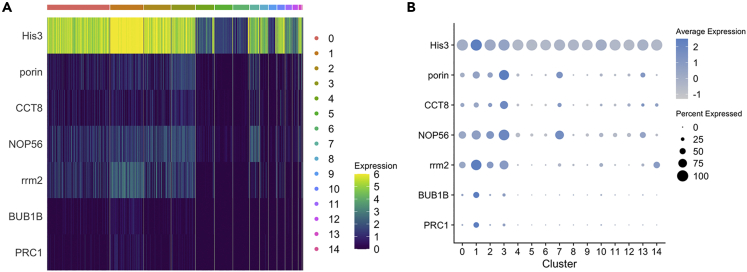
Expression levels and cluster distribution for selective HPT-specific transcripts (A) Heatmap and (B) dotplots.

As mentioned above, one cluster originating from the circulating hemocyte sample (CL11) was located close to this left cloud (Figure 1C) and was judged to represent hemocytes that have recently been released from the tissue. More than 94% of these CL11 cells expressed transglutaminase 1 (TGA1), which we have shown to be expressed exclusively in a minor proportion of hemocytes of the SGC morphology (Junkunlo et al., 2020). TGA1 is also known to be highly expressed in HPT cells and is of high importance in regulating the extracellular matrix structure and release of hemocytes into circulation (Junkunlo et al., 2016; Sirikharin et al., 2017). We used the TGA1 and one transcript encoding a hemolectin/hemocytin homolog (Hml) to identify more differentiated cells in the major HPT clusters. We found a clear expression gradient of TGA1 ranging from CL3<CL1<CL2 to CL0 (Figures 3D, 4A, and S4). Hemolectin (Hml) is used as a plasmatocyte marker in Drosophila hematopoiesis. A new study of hemocyte markers in the crayfish *Cherax quadricarinatus* demonstrated that Hml is expressed highly in HPT cells and to some extent in the semigranular cell population (Zhu et al., 2022). Furthermore, in recent single-cell transcript mapping in Drosophila, Hml was detected at low levels in prohemocytes and then showed increased expression in different subpopulations of plasmatocytes (Cho et al., 2020). We found a similar gradient of Hml expression, with the highest expression in circulating CL11 cells followed by CL0 and CL2 cells. Together, CL0 and CL2 constitute 55% of the total HPT cells in the main left cloud, and we consider these cells to be more differentiated than other cells in this cloud. It is likely that the majority of these cells together represent cells termed type 3 in the study by Chaga et al. (1995) (Figure 3B). Type 3 cells are the most easily liberated cells from the HPT, and they similarly constitute approximately 55% of the total number of cells (Chaga et al., 1995). Within the circulating hemocyte clusters in the right cloud, TGA1 and Hml expression levels were considerably lower and were detected only in minor proportions of the cells in CL4–CL6, CL9, and CL12 (Figure S4). Earlier, we showed that TGA1 mRNA is only detected in a subpopulation of the SGC fraction (Junkunlo et al., 2020); thus, these cells seem to be localized to CL11.

The increasing gradient of Hml and TGA1 expression in HPT indicates a differentiation pathway toward CL0 + CL2 in the HPT. As shown in Figure 2A, mitosis-associated transcripts were clearly specific to the HPT cloud, and the highest expression levels were found in in CL1 followed by CL3. Specific transcripts similar to mitosis-regulating genes, such as ASPM, tpx2, PRC1, BUB1B, rrm2, comp45551_c0, comp45345_c0, and comp48448_c0 (Figures 2A, S3A, 4A and 4B), were found to dominate in CL1. One of the most highly upregulated transcripts in CL1 was histone H3. As part of the nucleosome, this transcript is present in all cells but was clearly much higher in CL1, which may indicate a specific need in these mitotic cells (Figures 4A, 4B, and S4) (Luo et al., 2010). Thus, it is apparent that cells in CL1 and CL3 are highly mitotic, and may correspond to the type 2 cells (early precursor cells) according to Chaga’s terminology (Chaga et al., 1995). Apart from CL1 and other HPT clusters, CL3 was characterized by higher expression of a porin/VDAC-related gene. Porin/VDAC, a voltage-dependent anion channel, is involved in mitochondrial membrane permeabilization and mitochondrial remodeling processes that might lead to apoptosis or autophagy (Chen et al., 2011; Park et al., 2010), processes that both are ongoing in crayfish HPT (Lin et al., 2011). In addition, a CCT8-like transcript was dominant in CL3, which then gradually decreased in expression during the differentiation pathway (Figures 4A, 4B, and S4). CCT8 is part of the chaperone containing T-complex Theta, which assists in protecting stem cells from aggregating proteins and proteomic stress in plants (Llamas et al., 2021) and animals (Noormohammadi et al., 2016), and thus may be important in clearing up defective proteins that can be caused by high ROS production in the proliferating stem cells with high mitochondrial density (Noormohammadi et al., 2016). Tentative stem cells (type 1 in Figure 3B) are characterized by a high number of mitochondria per cell. During differentiation, the number of mitochondria per cell decreases. This reduction may occur through self-destruction by mitophagy, suggesting that CL3 represents a first step toward more differentiated cells. Another transcript specifically characterizing the HPT, and exhibiting the highest expression in CL3 was NOP56, a nucleolar protein involved in biogenesis of the 60S ribosomal subunit (Figures 4A and 4B).

### Iron-associated cells in the HPT

**Figure 5:**
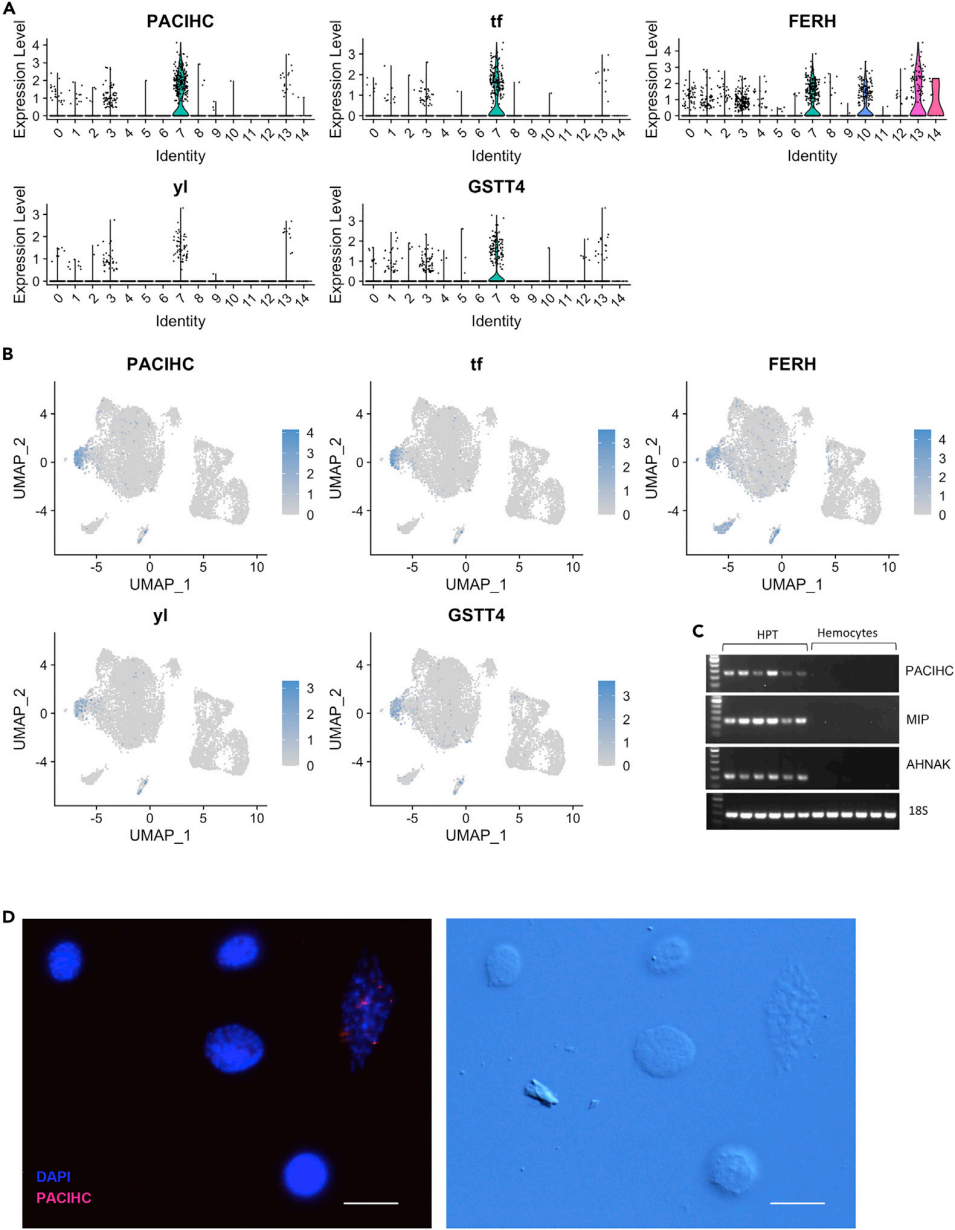
Marker transcripts in CL7, and validation of specific markers in HPT cells. (A) Violin plot and (B) UMAP for transcripts specifically expressed in CL7. (C) RT-PCR showing expression of PACIHC, MIP, and AHNAK in HPT cells (n = 6) and hemocytes (n = 6) respectively. (D) RNA-FISH using a QuantiGene ViewRNA probe linked to Alexa Fluor 546 showing mRNA expression of PACIHC in isolated HPT cells (left), and differential interference contrast of these cells (right). The bar indicates 20 μm in length.

We then further characterized CL7 and found the expression of several genes associated with iron homeostasis. Specific and unique for this cluster was a transferrin (tf) transcript that was present in approximately 40% of the cells (Figures 5A and 5B). An additional transferrin domain-containing transcript distinguishing CL7 from all other clusters except a few cells in CL13 was the transcript for the Pacifastin heavy chain subunit (PACIHC) (Liang et al., 1997), which contains three transferrin domains and exhibits high similarity to melanotransferrin in vertebrates (Suryo Rahmanto et al., 2012). We also assessed the expression of this transcript in six different individual animals by RT-PCR, and confirmed that the expression was strictly restricted to the HPT cells (Figure 5C). To reveal the cell type in which the transcript of PACIHC was expressed, we analyzed isolated HPT, including APC cells by RNA-FISH, and concluded that these cells had a large nucleus characteristic of stem cell morphology (Figures 3B and 5D).

Expression of the iron storage protein ferritin (FERH), which was previously characterized from *P. leniusculus* (Huang et al., 1996), was also a feature associated with CL7. This finding may indicate a need for protection against iron overload, which is highly likely to occur during mitochondrial turnover (Figures 5A and 5B). In addition, some unknown transcripts were highly and specifically expressed in CL7, revealing the distinct properties of cells in this cluster and indicating that cells in this cluster may exhibit precise properties (Figure S5). We previously described and characterized the clotting protein in detail, and this protein is a vitellogenin-like large lipoprotein and constitutes an important component of the extracellular matrix in HPT (Junkunlo et al., 2018) and of course also as a component of the coagulation system (Cerenius and Söderhäll, 2011; Hall et al., 1999). We currently observed that a minor portion of the CL7 cells expressed a putative vitellogenin receptor (yl), indicating a close association with the clotting protein. It is possible that these yl cells are tightly attached to the extracellular matrix as the type 1 precursor-containing cells according to Chaga et al. (1995). Because the cells in CL7 have so many proteins/transcripts directly associated with iron homeostasis, it is reasonable to propose that these cells control and regulate mitochondrial turnover and thus mitophagy. However, this hypothesis needs to be tested in functional studies.

### Other specific HPT cell types

**Figure 6:**
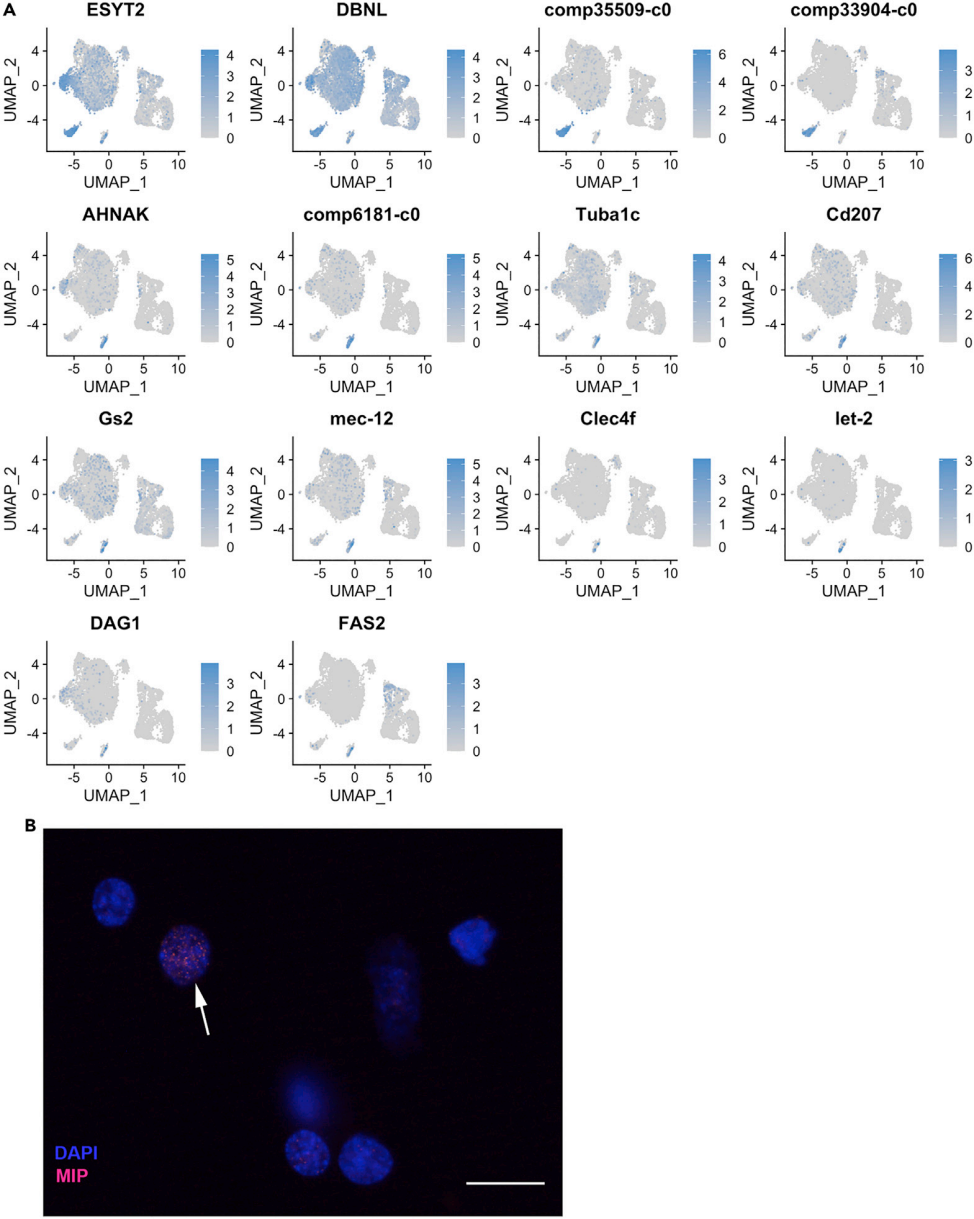
Characterization of the separated HPT clusters CL10 and CL13 (A) UMAPs showing expression distribution of transcripts characterizing CL10 (top 4) and CL13 (lower 10). (B) RNA-FISH using a QuantiGene ViewRNA probe linked to Alexa Fluor 546 showing mRNA expression of MIP (comp6181_c0) in isolated HPT cells. The bar indicates 20 μm in length.

The HPT sample clustered into 9 different clusters, and two of these were clearly separated from the main left cloud namely CL10 and CL13 (Figures 1C and 6A).

The majority (90%–100%) of CL10 cells were characterized by high expression of ESYT2, and a Debrin-like transcript (DBNL), both of which may play roles in cytoskeleton remodeling or a function in endocytosis (Jean et al., 2010; Rocha-Perugini et al., 2017) (Figure 6A). One transcript, MYO18A, was specific to CL10 but not in all cells, and only approximately 20% of the cells in this cluster contained this transcript. In vertebrates, MYO18A is involved in cytoskeleton-dependent Golgi membrane trafficking and was recently found to be important for the regulation of B cell homeostasis in mice (Cheung et al., 2021). A role for this protein in invertebrates remains to be reported. However, it is certainly of interest to elucidate a possible function of the product of MYO18A in crayfish, especially given that a separate portion of CL10 cells contained this transcript/protein. Moreover, a Hpgds-like gene similar to the hematopoietic prostaglandin D synthase was unique for CL10. Interestingly, the most highly expressed transcript in all CL10 cells that was restricted to this cluster was comp35509_c0, which encodes a previously undescribed peptide with the sequence *MTQAVVKVILCCLRVLP*LAPPRPEVPINTNDSTSNCKDYRITYLPGIF (putative signal peptide in italics). The function of this peptide is still unknown (Figure 6A).

To date, we have been unable to detect any cells with expression patterns similar to the posterior signaling center (PSC) in the Drosophila lymph gland, which is known to have a regulatory function in the hematopoietic stem cell niche, in crustacean HPTs (Banerjee et al., 2019; Girard et al., 2021; Mandal et al., 2007). In the present single-cell analysis, we could not identify any of the key PSC-defining transcripts. However, in CL10, a PVF transcript not previously described was detected in a large portion (approximately 82%) of the cells (comp33904_c0). In Drosophila, PVF1 is secreted from the PSC and regulates the proliferation in the cortical zone by binding to a PVR (a PDGF/VEGF receptor) (Girard et al., 2021). This finding is interesting given that PVF/PVR signaling in this crustacean regulates the activity of extracellular TGase activity. As a result, this signaling appears to block cell/hemocyte release from the HPT (Junkunlo et al., 2017). Notably, very few cells in the HPT and a small number of circulating cells expressed PVR as detected by ISH (Junkunlo et al., 2017). In this single-cell study, PVR-expressing cells were exclusively detected in a minor part of CL12 (Flt1, 11%, see below). As a consequence, we believe that these cells originated from the HPT sample (Figure 1D). These results may suggest that something similar to a PSC or a partial PSC may be present in crustaceans. However, our limited data do not provide conclusive evidence for a PSC-like cluster in crayfish HPT, but may justify further investigations.

A second small distinct cluster (CL13, Figure 1C) exhibited several highly differentially expressed and also unique transcripts. Among these, we detected the melanization interacting protein (MIP) (Söderhäll et al., 2009) in >90% of the cells (comp6181_c0). This protein contains a fibrinogen-like domain, is secreted into plasma, and acts as a regulator of the melanization reaction (Söderhäll et al., 2009). The MIP transcript was also confirmed to be exclusively expressed in the HPT using samples of hemocytes and HPT from six different individuals (Figure 5C) and could be detected by RNA-FISH in HPT cells (Figure 6B).

In addition, a C-type lectin of the CLEC4 family as well as a C-type lectin receptor Langerin-like (Cd207) was also unique or highly expressed in CL13 together with the tubulin transcripts Tuba1c, Mec-12, and two transcripts encoding extracellular matrix proteins Let_2 (collagen type IV) and DAG1 (dystroglycan) (Figure 6A).

Other transcripts highly specific in this CL13 include the neuroblast differentiation-associated protein AHNAK (82% of the cells), glutamine synthase 2 (40% of the cells), and a Fasciclin-2-like transcript (25% of the cells) (Figure 6A). As shown in Figure 5C, we also validated the restricted expression of AHNAK in the HPT cells. It is interesting that these three transcripts, AHNAK, FAS2, and GS2, may be associated with neural development and are present in this highly separated cluster (Figure 1C). Therefore, cells CL13 would be of interest to study further to find out if they are linked to the process which was previously described to involve cells from the hemolymph that are converted to neurons in the brain (Benton et al., 2014, 2022).

While CL0 and CL2 were considered as more differentiated precursor cells with high expression of TGA1 and Hml, the small CL8 was instead characterized by downregulation of most transcripts and in particular transcripts encoding ribosomal proteins, (Figure S6A). Due to the lack of clear marker transcripts, it is difficult to judge what these cells (CL8) represent. These cells could be quiescent stem cells or they may constitute a population of cells that is about to undergo differentiation, and the downregulation indicates a remodeling of the cell transcriptome that may precede differentiation and the formation of new transcripts for determined cells (Antolović et al., 2019). As shown in Figure S6B, transcripts encoding ribosomal proteins were also expressed at low levels in circulating hemocytes in CL5, CL6, and CL9, cells, which are highly differentiated hemocytes. When comparing transcriptomes of HPT cells and mature circulating hemocytes, considerable differences are noted, indicating a need for specific RNA decay or remodeling mechanisms, a process required for cell differentiation (Li et al., 2015; Lou et al., 2015). CL8 did not show any specific marker transcripts; therefore, it is difficult to determine whether these cells were more differentiated and on the verge of mature hemocytes, or whether they instead represented quiescent undifferentiated stem cells.

### Cell clusters among the circulating hemocytes

The circulating hemocytes in the integrated analysis were separated into six different clusters (Figure 1C). One of these (CL11) was close to the HPT cloud, and clear marker transcripts of the cells in this cluster included Hml, TGA1, and Duox (Figures 3D and S4). The localization of CL11 close to the HPT cloud indicates that these cells had a transcriptome profile similar to HPT cells, as mentioned above. We also consider the CL11 cells to be part of the SGC fraction given that almost all cells (≈97%) expressed TGA1, which is only expressed in SGCs (Junkunlo et al., 2020). The SGC cell population is also a specific type of hemocyte that is involved in both phagocytosis and coagulation, and these cells also contain Hml. Interestingly, Hml is associated with both phagocytosis and coagulation in insects (Goto et al., 2001, 2003). CL11 consists of a rather small fraction of the circulating hemocytes (≈9% in this sample). Consistent with this result, phagocytotic activity in *P. leniusculus* has been shown to occur in a very small proportion of the SG cell fraction (Söderhäll et al., 1986). This type of phagocytic cell is quite rare in crayfish. Of interest here is that a high Hml expression was detected in a hemocyte type described as prohemocytes in a scRNA experiment in the shrimp *Marsupenaeus japonicus (*Koiwai et al., 2021). This finding is in contrast to Drosophila larvae, where phagocytotic plasmatocytes are by far the dominant hemocyte type, which may be because of their important role in metamorphosis during the pupal stage. In addition, the Duox-like transcript dominating in CL11 provides further support that this cluster contains a large number of phagocytic cells.

**Figure 7:**
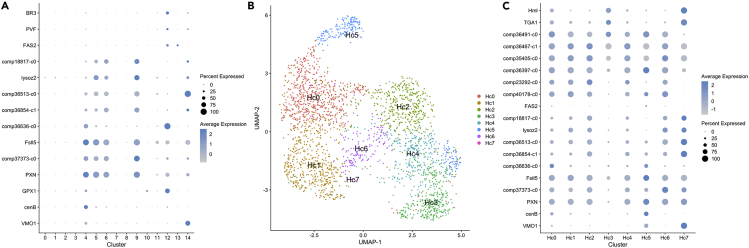
Comparison of hemocyte clusters in the integrated and single analysis (A) Dotplots showing expression levels and cluster distribution for selected hemocyte transcripts in the integrated HPT and hemocyte DEG analysis. (B) UMAP plot for the hemocytes sample showing the different clusters obtained when the hemocyte sample was analyzed separately. (C) Dotplots showing expression levels and cluster distribution for selected hemocyte transcript in the hemocyte DEG analysis.

Another small hemocyte cluster (CL12, ≈9% of the hemocytes) expressed a C-type lectin transcript (comp36636_c0, similar to the GAFS01005145.1 in *Pontastacus leptodactylus*) in nearly all cells. This transcript, as well as a glutathione peroxidase (GPX1-like), was also found in the larger hemocyte CL4, although in a minor proportion of the cells and at a lower expression level compared to that occurring in the CL12 cells (Figure 7A). In addition to the C-type lectin and GPX1 present in CL12, a unique expression of three PDGF- and VEGF-related factors (PVF1 ASU10867.1, BR3, and a previously unknown transcript comp34335_c0) together with a PDGF and VEGF receptor (FLT1) was noted in a minor proportion of the cells.

Furthermore, a small portion of the cells in CL12 expressed a FAT1-related transcript that is similar to the protocadherin in *Homarus americanus* (Polinski et al., 2021). This transcript produces a cell adhesion protein that is involved in neurodevelopmental processes in humans (Mancini et al., 2020), and this transcript was not detected in any other clusters. A fasciclin-2-like transcript (FAS2) was present in approximately 34% of the CL12 cells, and this transcript, which is also associated to neuronal recognition, was also found in CL13 of the HPT in addition to its presence in CL12 as mentioned above (Figures 6A and 7A).

**Figure 8:**
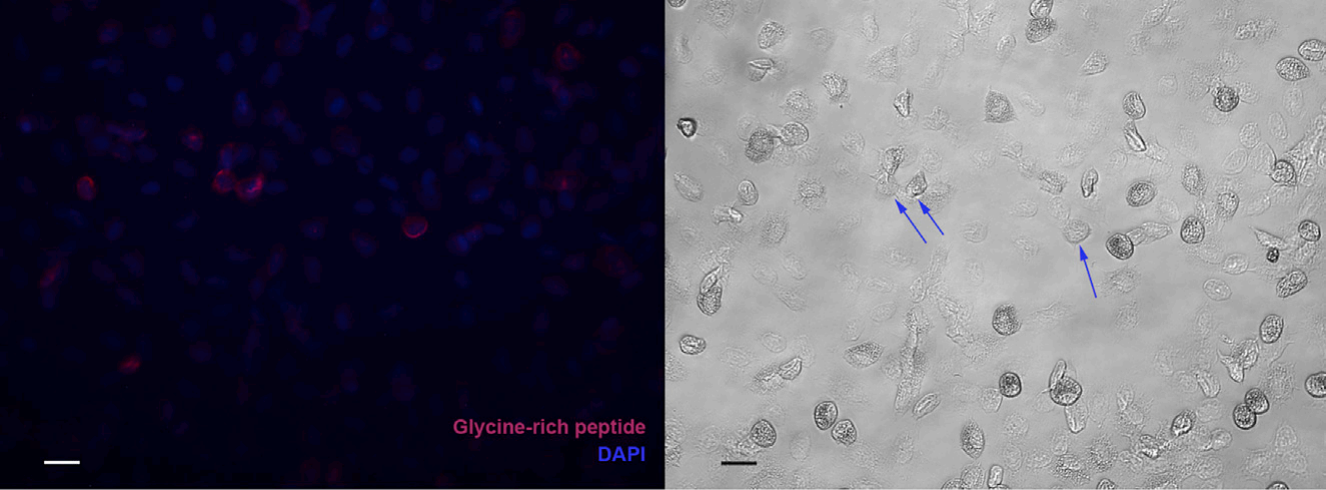
Expression of a glycine-rich peptide in isolated hemocytes RNA-FISH of hemocytes using a QuantiGene ViewRNA probe linked to Alexa Fluor 647 designed to recognize mRNA encoding a glycine-rich peptide (comp18817_c0), nuclei stained with Dapi (left). Light microscopy of the same field, some of the peptide expressing cells are indicated by blue arrows. The bar indicates 20 μm in length.

The majority of the circulating hemocytes were present in CL4-6 and CL9, and all these cells expressed our previously well-characterized immune response-associated genes to various degree (Figure 7A, crustins in Figure S3). While CL5, 6, and 9 had rather similar transcript profiles, we could detect some definite transcripts in CL4, namely a vitelline membrane outer layer 1 homolog (VMO1) (Sricharoen et al., 2005; Zhu et al., 2022) and cenB, an endoglucanase. As mentioned above, a C-type lectin was also highly expressed in CL4 together with CL12 (Figure 7A). The proPO transcript (comp37373_c0) together with masquerade (Huang et al., 2000) (comp36854_c0), a lysozyme (lysoz2), astacidin2 an AMP (comp36513_c0) (Jiravanichpaisal et al., 2007a), and a glycine-rich peptide (comp18817_c0) (Ekblom et al., 2021; Sricharoen et al., 2005) exhibited increased expression from CL5 to CL6 with the highest levels of expression noted in CL9. As shown in Figure 8, expression of the glycine-rich peptide was observed in some but not all of the cells that exhibited GC-like morphology. On the other hand, similar expression patterns were noted for the different crustins, with the exception of crustin-36397, which was highly expressed in CL4 and in CL12 (Figures S3A and S3B). Taken together, we identified at least four distinct cell types among the circulating hemocytes (CL11, CL12, CL4, and CL5+6 + 9) in the integrated single-cell analysis.

**Figure 9:**
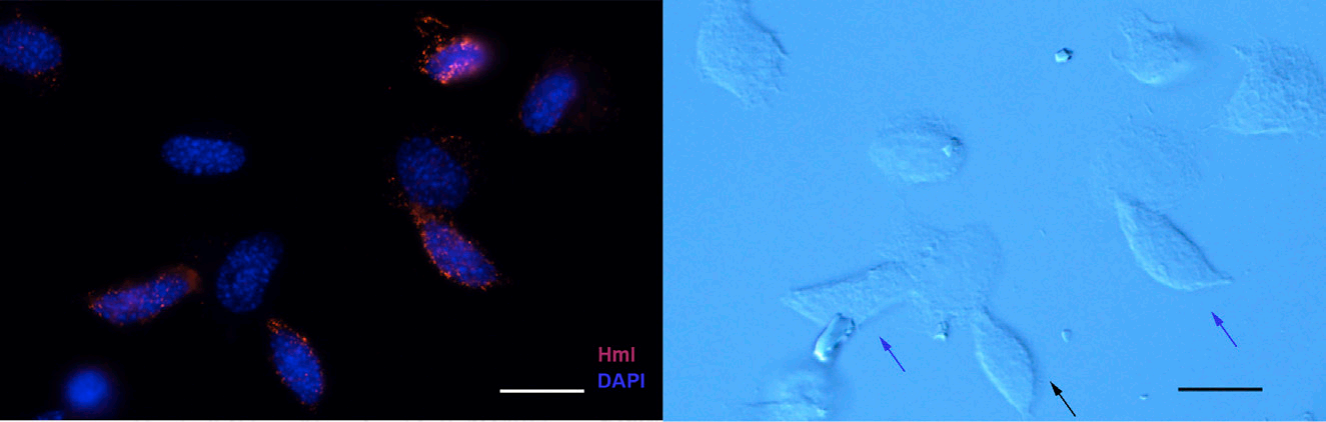
Expression of hemolectin in isolated hemocytes RNA-FISH of hemocytes using a QuantiGene ViewRNA probe linked to Alexa Fluor 546 designed to recognize mRNA encoding Hemolectin (Hml), nuclei stained with Dapi (left). Differential interference microscopy of the same field, typical semigranular cells are indicated by blue arrows. The bar indicates 20 μm in length.

To confirm the specific cell types identified above, we reanalyzed the hemocyte samples as a single sample. The cells clustered into six different clusters (Figure 7B) (Hc-CL7 consisted of only 5 cells, and for this reason, it is not discussed further). All immune-related genes illustrated in Figures 3, 7A, and S3 were plotted onto these new hemocyte clusters (Figure 7C). We concluded that the Hml and TGA1 transcripts were clearly highly expressed in a separate cluster Hc-CL3, while VMO1 and cenB together were highly expressed in Hc-CL5, which was also the case for the C-type lectin (comp36636_c0). As concluded above, Hml was mainly expressed in cells with semigranular morphology, as shown by RNA-FISH in Figure 9.

These results confirm the presence of two specific cell types. Interestingly, Hc-CL3 cells did not express proPO, astacidin2, or masquerade. On the other hand, Hc-CL5 cells expressed some unique transcripts, including FAS2, and a kallikrein-related protease KLK6, in a minor proportion of the cell population.

### Conclusions

The main and important results of the scRNA analysis of HPT cells and hemocytes in a crustacean, the freshwater crayfish, *P. leniusculus*, clearly demonstrated that a very high number of transposons are present in this species. Whether this level of TEs is significant remains to be elucidated. Given that no genome is available for this crayfish species, our transcriptome studies are limited, but it was possible to clearly demonstrate that the circulating cells, namely, the hemocytes, contain several different subtypes and in this sense are even more diverse than previously thought. However, it should be emphasized that our early studies of hemocyte types clearly showed that each class of hemocyte types consists of several subtypes (Junkunlo et al., 2017, 2020), and now we can clearly demonstrate that this is the case. We have summarized characteristics of each cell cluster below in Table 1.

**Table 1:** Summary of the characterization of the different cell clusters, as described in the text

Cluster	HPT/Hc cell number	Signature transcripts	Comment
CL0	2292/22	TGA1 Hml	TGA1 is a crosslinking enzyme important in coagulation and regulation of the extracellular matrix in the HPT. Hml is shown to be a marker for phagocytes in insects. There is a gradient of these transcripts in the HPT from immature cells to more developed precursors.
CL1	1199/29	His3 ASPM comp45345_c0 (regulator of cytokinesis 1-like) comp45551_c0 (putative protein MIS12 homolog) tpx_2b	All these transcripts are associated with mitosis.
CL2	988/8	Actin5c TGA1 Hml	See CL0. The proportions of transcripts differ between CL0 and CL2, and also for several unknown transcripts.
CL3	884/2	Porin/VDAC CCT8 NOP56	Porin/VDAC may be a sign of high number of mitochondria. CCT8 a chaperon may protect cells from stress such as high ROS production. NOP56 is involved in ribosomal subunit 60S biogenesis.
CL4	66/609	VMO1 cenB crustin-36397 glutathione peroxidase (GPX1-like)	VMO1 is involved in vitellogenesis and its function in immunity is unknown. cenB is an endoglucanase. crustin-36397 is a putative antimicrobial peptide. GPX1 protect cells from oxidative stress.
CL5	7/657	Crustin 1-3 proPO astacidin2 Gly-rich peptide masquerade	Crustin 1-3 , astacidin2 and the gly-rich peptide are antimicrobial peptides. proPO and masquerade are components of the melanization reaction.
CL6	46/548	Crustin 1-3 proPO astacidin2 Gly-rich peptide masquerade	Crustin 1-3 , astacidin2 and the gly-rich peptide are antimicrobial peptides. proPO and masquerade are components of the melanization reaction.
CL7	349/0	PACIHC Transferrin FERH yl	PACIHC, transferrin and FERH are involved in iron homeostasis. PACIHC is also part of the protease inhibitor pacifastin. yl is a putative vitellogenin receptor.
CL8	289/10	No specific markers Downregulation of transcripts encoding ribosomal proteins	
CL9	51/247	Crustin 1-3 proPO astacidin2 Gly-rich peptide masquerade	Crustin 1-3 , astacidin2 and the gly-rich peptide are antimicrobial peptides. proPO and masquerade are components of the melanization reaction.
CL10	277/2	ESYT2 PDGF/VEGF-domain (PVF-like factor) Debrin-like MYO18A comp35509_c0	ESYT2, MYO18 and Debrin may be involved in cytoskeleton remodelling or endocytosis. PDGF/VEGF-domain proteins are growth factors and likely involved in the differentiation process. comp35509_c0, a small peptide of unknown function.
CL11	3/235	Hml TGA1 Duox	Hml and Duox are involved in phagocytosis in insects. TGA1 is the crosslinking protein in the clotting reaction.
CL12	6/195	comp36636_c0 (C-type lectin) glutathione peroxidase (GPX1-like) crustin-36397 PVF1 (PDGF/VEGF-domain) BR3 (PDGF/VEGF-domain) comp34335_c0 (PDGF/VEGF-domain) FAT1-related (protocadherin) FAS2 (Fasciclin-2-like)	comp36636_c0 a C-type lectin usually involved in pattern recognition. GPX1 protect cells from oxidative stress. crustin-36397 is a putative antimicrobial peptide. PVF1, BBR3 and comp34335_c0 are all PDGF/VEGF-domain proteins and likely involved in regulation of growth and development. FAT1-related and FAS2 are cell adhesion proteins and could be involved in neuronal development and/or immunity.
CL13	121/0	MIP AHNAK FAS2 (Fasciclin-2-like) Glutamine synthase 2 (GS2) Clec4f Cd207-like (langerin) DAG1 (dystroglycan) Let_2 (collagen type IV) Tuba1c Mec-12	MIP a melanization regulator AHNAK, FAS2 and GS2 may be linked to neural development. Clec4f is a C-type lectin. Cd207 is a putative C-type lectin receptor. Dag1 and Let_2 encode putative extracellular matrix proteins. Mec-12 and Tuba1c are tubulin encoding transcripts.

Transcripts with high or unique expression are named as signature transcripts for their respective clusters. The number of cells from each sample (HPT or hemocytes) is indicated in the second left column.

Some very specific cell types could be found, and many cells of CL11 and CL12 belong to the SG cell class of hemocytes with distinct transcripts, such as TGA1 and Hml. With regard to SG cells, we previously found that after separation in Percoll gradients, this class of hemocytes consisted of several subtypes, some of which were most likely on the way to become granular cells. This finding was confirmed in this study. In particular, CL5, CL6, and CL9 most likely represent different stages of GCs.

An exciting discovery was that one cluster (CL7) consisted of several transcripts that we have previously described and characterized in this crayfish species. These transcripts are involved in iron homeostasis and form a strict separate cluster. Iron homeostasis is obviously very important during hematopoiesis in vertebrates, due to the continuous synthesis of red blood cells with hemoglobin. Crustaceans (as well as insects) do not have these cells, which means that iron homeostasis during hematopoiesis has other functions. One possible function may be to regulate the iron concentration in cells during mitochondrial metabolism. This organelle is most frequently found in the least differentiated stem cells (see TEM figures of type 1 in Chaga et al., 1995). During differentiation, the number of mitochondria per cell decreases, and this process could involve mitophagy. Thus, CL7 may be important in regulating and controlling mitochondrial turnover; hence, this type of cell may also be important for hematopoiesis also in other animals. Another very specific cell type includes the cells found in CL13 containing the MIP, which is known to be a regulator of the melanization reaction in arthropods and many other invertebrates. Thus, it is likely that these cells are key regulators for releasing of MIP protein. It was not possible to draw a definite and distinct pathway for differentiation from precursor hemocyte types to mature hemocytes, but some new and distinct hemocyte types were discovered and described in this study. Some of these new hemocyte types are involved in the differentiation pathway, and some are possibly implicated in neurogenesis and control of innate immune reactions.

### Limitations of the study

A previously mentioned limitation of the current study is that it was performed at the transcript level, given that a genomic reference for *P. leniusculus* is currently lacking. The sequencing depth is thus divided between a large number of features, compared to what would have been achieved at the genome level. However, efforts are currently underway to sequence and annotate the genome, which could potentially be used to reanalyze these data in the future and further explore these reported findings.

It is well known that hemocytes from crayfish are very sensitive and easily aggregate and lyse, which we believe is a possible explanation for the fact that significantly fewer hemocytes regained quality control compared to the much more stable cells from HPT (6585 HPT cells vs. 2546 hemocytes). An ideal method for single-cell analysis could therefore be to manually pick out hemocytes for analysis in a 384-well plate. This can of course mean that we have lost certain cell types that occur in very low frequency.

## STAR★Methods

### Key resources table

**Table:** 

REAGENT or RESOURCE	SOURCE	IDENTIFIER
**Antibodies**		

Anti-BrdU mouse monoclonal	Sigma	Cat# B8434, RRID:AB_476811
Anti-mouse-FITC	Sigma	Cat# F3008, RRID:AB_259513

**Chemicals, peptides, and recombinant proteins**		

Collagenase Type I	Sigma	C0130
Collagenase Type IV	Sigma	C5138
RNA Trizol LS Reagent	Thermo Fisher	10296010
PureLink® RNA Mini Kit	Thermo Fisher	12183025
PrimeScript 1st strand cDNA Synthesis Kit	Takara	RR047A
Phusion™ High Fidelity DNA Polymerase	Thermo Fisher	F530S
SYBR™ Safe DNA Gel Stain	Invitrogen	S33102
Probe PACIHC (Alexa Fluor 546)	ThermoFisher	CVX-01 ID:VPDJXJY
Probe Peptide 18817 (Alexa Fluor 647)	ThermoFisher	CVX-01 ID:VP2W7RT
Probe Hml (Alexa Fluor 546)	ThermoFisher	CVX-01 ID:VPCE3Y2
Probe Control neg. (Alexa Fluor 546)	ThermoFisher	DapB probe
Formalin contains 10–15% methanol as stabilizer, 37 wt. % in H_2_O	Sigma	F1635
Paraformaldehyde 4% in PBS	ThermoScientific	J19943.K2
BrdU	Sigma	B5002
Pepsin	Sigma	P7000
BSA	Sigma	A8531
Vectashield	Vector laboratories	H-1000
Propidium iodide	Sigma	P4170
Prolong® Gold Antifade Reagent	ThermoFisher	P36930

**Critical commercial assays**		

Chromium Single Cell 3′ reagent kit v3	10x Genommics	PN-1000075/PN- 120262
QuantiGene ViewRNA ISH Cell assay	ThermoFisher	QVC0001

**Deposited data**		

Accession number for raw data	Array Express	E-MTAB-11745

**Experimental models: Organisms/strains**		

*Pacifastacus leniusculus* Male intermolt	Lake Erken Sweden	Tax ID:6020

**Oligonucleotides**		

Primer PACIHC forward: GATGGCTGGTACAAGTTTA	ThermoFisher	n/a
Primer PACIHC reverse: TGACTAAACGAGTGATGTT	ThermoFisher	n/a
Primer AHNAK forward:	ThermoFisher	n/a
Primer AHNAK reverse: AATGTCTACTGCAACTGAGG	ThermoFisher	n/a
Primer MIP forward: AGGTACACTCTCATCTACCC	ThermoFisher	n/a
Primer MIP reverse TCAAGATAGTCTGCAGTGTG	ThermoFisher	n/a
Primer 18S forward: AGTTTCAGCACATCCTGCCTTCCCTTCAGA	ThermoFisher	n/a
Primer 18S reverse: GACCACGATGTGCACGAATCTTCTTCATGC	ThermoFisher	n/a

**Software and algorithms**		

All original code has been deposited at https://github.com/NBISweden/SMS-21-5568-cray	n/a	n/a

### Resource availability

#### Lead contact

Further information and requests for resources and reagents should be directed to and will be fulfilled by the lead contact, Irene Söderhäll (Irene.Soderhall@ebc.uu.se).

#### Materials availability

The study did not generate any new reagents.

### Experimental model and subject details

Freshwater crayfish *Pacifastacus leniusculus* (adult intermolt males, weight 30–35 g) were obtained from lake Erken (located in east-central Sweden near the Baltic coast (59.8 N 18.6 E). The animals were maintained in the crayfish facility at department of Organismal Biology, Uppsala University, in running tap water at 10–12°C, in a 12:12 light:dark cycle. The water was aerated with plastic tubing and ceramic oxygen stones. The animals were fed once a week.

### Method details

#### Tissue preparation and BrdU labeling and detection

The crayfish were injected with 10 μL/g fresh weight of 50 mM BrdU dissolved in crayfish saline (CFS:0.2 M NaCl, 5.4 mM KCl, 10 mM CaCl2.2H2O, 2.6 mM MgCl2.6H2O, 2 mM NaHCO3, pH 6.8). At 3 h post injection the stomach covered with the HPT was dissected from crayfish and immediately fixed in 4% paraformaldehyde in PBS and kept at 4°C overnight. Then the HPT was carefully removed from the stomach, dehydrated through an ethanol series 50%, 70%, 90%, 100%, and xylene, and finally embedded into paraffin blocks. The HPT was then sectioned vertical sections from anterior to posterior with thickness of 10 mM and the sections were placed on MENZEL Super frost glass slides for further processing. After deparaffination and rehydration the slides were washed with CPBS-TB (CPBS:10 mM Na_2_HPO_4_, 10 mM KH_2_PO_4_, 0.15 M NaCl, 10 μM CaCl_2_, 10 μM MnCl_2,_ 2.7 μM KCl; pH 6.8, containing 0.5% Tween 20 and 0.5% BSA). The slides were then incubated in 2 N HCl containing 0.mg/mL pepsin for 30 min at 30°C. After washing 5 times with CPBS-TB, the slides were incubated in anti-BrdU mouse monoclonal antibody (Sigma) dissolved in CPBS-TB for 30 min at RT, followed by secondary antibody FITC-labeled anti-mouse IgG (Sigma) in CPBS-TB for 30 min at RT. Finally, the nuclei were stained with propidium iodide (10 μg/mL) and mounted in Vectashield.

#### Cell preparation for scRNA sequencing

Each Hpt and APC was dissected from the crayfish, and digested into single cells by incubation in 300 μL of 0.1% collagenase (Type I), 0.1% collagenase (Type IV) in CPBS at room temperature for 20 min on a rotating plate. After collagenase treatment the tissue was gently passed 10–20 times through a Pasteur pipette and centrifuged at 800 *g* for 5 min to remove the collagenase solution. The pellet was washed once in 1 mL 0.15 M NaCl with 0.04% BSA by centrifugation at 800 x *g* for 5 min, suspended in 1 mL 0.15 M NaCl with 0.04% BSA and then filtered through a 40 mm cell strainer. Isolated cells from four animals were pooled to the HPT sample used for scRNA-seq.

Hemocytes were collected by bleeding 2 mL in a 1:1 volume with anti-coagulant solution (0.14 M NaCl, 0.1 M glucose, 30 mM trisodium citrate, 26 mM citric acid, 10 mM EDTA, pH 4.6) using a 18 G needle (BD microlane). Samples were centrifuged for 5 min at 800 × *g*. The supernatant was discarded, and the cell pellet washed in 2 mL 0.15 M NaCl. The washing was repeated two more times, and finally the hemocytes were suspended in 1 mL 0.15 M NaCl with 0.04% BSA and used as hemocyte sample for scRNA-seq. The cell numbers in both samples were determined with a hemocytometer.

#### scRNAseq

For each sample (HPT + APC) or hemocytes, 10000 cells were delivered to the SNP&SEQ Technology Plat-form in Uppsala for single cell RNA-sequencing, using 2 lanes on a NovaSeq SP flowcell. Sequencing libraries were prepared using Chromium Single Cell 3′ reagent kit v3 (cat# 1000075/1000073/120262, 10xGenomics) according to the manufacturer’ protocol (CG000183 Single Cell 3′ reagent Kit User Guide, v3 chemistry, 10x Genommics). The details for sequencing were as follows: 28 + 8+0 + 91 bp read lenght, NovaSeq 6000 system, SP flowcell and v1 sequencing chemistry. A sequencing library for the phage PhiX was included as 1% spike-in in the sequencing run. The result files were delivered and stored at the Swedish National Infrastructure for Computing (SNIC) at [SNIC UPPMAX].

#### Processing of scRNAseq data

A custom transcript-to-gene mapping was created with the results of previously performed transcript annotations (including BLAST queries), known mitochondrion sequences as well as previously known transcript-to-gene mappings. Quantification of scRNA-seq data was performed using Alevin (Srivastava et al., 2019), the transcriptome and the and the custom transcript-to-gene mapping. Quality controls was performed using AlevinQC. [https://csoneson.github.io/alevinQC/].

#### scRNAseq data analysis

Bioinformatic analyses of the Alevin output was performed using the Seurat package (Hao et al., 2021) of the R programming language, with modifications and additions. Cell libraries were considered to be of low quality and filtered out if: (1) fewer than 10 features were detected; (2) they had a higher mitochondrial content than 30%; (3) they had a higher ribosomal content than 7.5%. Features present in fewer than 3 cells were excluded, as were mitochondrial, ribosomal and features related to heat shock. Feature counts were log-normalized with a scaling factor of 10000, followed by Z-score transformation for regression of confounding factors and downstream clustering.

The normalized data was subsequently processed using principal component analysis (PCA), from which the top 50 components were selected for downstream analyses. The Uniform Manifold Approximation & Projected (UMAP) (McInnes et al., 2018) non-linear method for dimensionality reduction was run on the top 50 principal components, yielding a final embedding of two dimensions. Unsupervised graph clustering using the Louvain method was run using a resolution parameter of 0.7, optimized to yield a coherent cluster distribution in the UMAP. Differential expression testing between clusters was performed using MAST. Differential expression testing between cases and controls for each previously defined cluster was performed using MAST (Finak et al., 2015).

All bioinformatic analyses have been put into a Nextflow-based workflow to facilitate reproducibility, which can be found on GitHub.

#### Fluorescent *in situ* hybridization

Hemocytes and HPT cells were isolated as above and attached to carefully washed and autoclaved coverslips in 24-well plates. After attachment the cells were fixed in 3.7% formalin in CFS for 10 min at room temperature. The formalin was removed and exchanged for 70% ethanol and the plates were stored at −20°C overnight. The plates were then used for fluorescent RNA *in situ* hybridization using the QuantiGene ViewRNA ISH Cell assay kit and procedure according to the manufacturer’s user manual. The cells were finally mounted in Prolong® Gold Antifade Reagent and analyzed using fluorescence microscopy with a Leica DM IL Led inverted (Figure 8), a Leica DM5500B upright microscope (Figures 5, 6, and 9).

#### RNA extraction and RT-PCR

Tissue samples from crayfish (HPT and APC) were collected and stored in 500 μL TRIzol (Thermo Fisher) at −80°C. For hemocytes, 0.5 mL of hemolymph was collected in an equal volume of anti-coagulant (0.14 M NaCl, 0.1 M glucose, 30 mM trisodium citrate, 26 mM citric acid, 10 mM EDTA, pH 4.6), and centrifuged for 5 min at 800 x g. The supernatant was discarded, and the pellet washed in 1 mL 0.15 M NaCl. The hemocytes were pelleted by centrifugation again, and the supernatant was removed, after which the hemocytes were stored in TRIzol in the same way as the other tissues.

Total RNA was extracted using the PureLink® RNA Mini Kit (Thermo Fisher). Briefly, the samples were thawed on ice, and homogenized with a tissue grinder, and by passing through a 0.4 mm needle. RNA was extracted with 100 μL chloroform (Supelco). The samples were incubated at room temperature for 3 min, and centrifuged at 12,000 *x g* for 15 min at 4°C. The aqueous phase was transferred to a new tube, and an equal volume of 70% ethanol was added. The samples were vortexed, and RNA washed using the Purelink® Kit. RNA was eluted in 20 μL RNase Free Water from the kit, and 0.5 μg of RNA was used to synthesize cDNA using the PrimeScript 1st strand cDNA Synthesis Kit (Takara). The cDNA was stored at −20°C until use.

The PCR reaction for each gene was prepared in 20 μL, using Phusion™ High Fidelity DNA Polymerase (Thermo Fisher), with the 5X PhusionTM HF Buffer and dNTP Mix provided by the manufacturer, according to their instructions, using 0.5 μL cDNA. The PCR program was 98C for 15 s, followed by 25-30 cycles of 98C for 30 s, 60C for 30 s, and 72C for 30 s, and one cycle of 72C for 5 min. The PCR products were separated on a 1.5% agarose gel stained with SYBR™ Safe DNA Gel Stain (Invitrogen) and visualized with FujiFilm Life Science Imaging Systems LAS4000.

### Quantification and statistical analysis section

Protein levels (LFQ = label-free quantitation) of crustin 1, crustin two and crustin 3 in HPT cells (n = 9), semigranular cells (SGC, n = 5) and granular cells (GC, n = 4) respectively were illustrated using GraphPad Prism 9 (Figure S3).

## Data Availability

Single-cell RNA-seq data have been deposited at ArrayExpress (https://www.ebi.ac.uk/arrayexpress/experiments/E-MTAB-11745) and are publicly available as of the date of publication. Accession numbers are listed in the key resources table. Microscopy data reported in this paper will be shared by the lead contact upon request. All original code has been deposited at https://github.com/NBISweden/SMS-21-5568-cray and is publicly available as of the date of publication. DOIs are listed in the key resources table. Any additional information required to reanalyze the data reported in this paper is available from the lead contact upon request.
