# Supplementary material for: Characterization of hemocytes and hematopoietic cells of a freshwater crayfish based on single-cell transcriptome analysis
Source: iScience. 2022 Aug 2;25(8):104850. doi: 10.1016/j.isci.2022.104850 (PMC9391574; doi:10.1016/j.isci.2022.104850)
Supplement: Document S1. Figure S1–S6 [file mmc1.pdf]

iScience, Volume *25*

Supplemental information

Characterization of hemocytes and hematopoietic cells of a freshwater crayfish based on single-cell transcriptome analysis

Irene Söderhäll, Erik Fasterius, Charlotta Ekblom, and Kenneth Söderhäll

Supplemental Figures

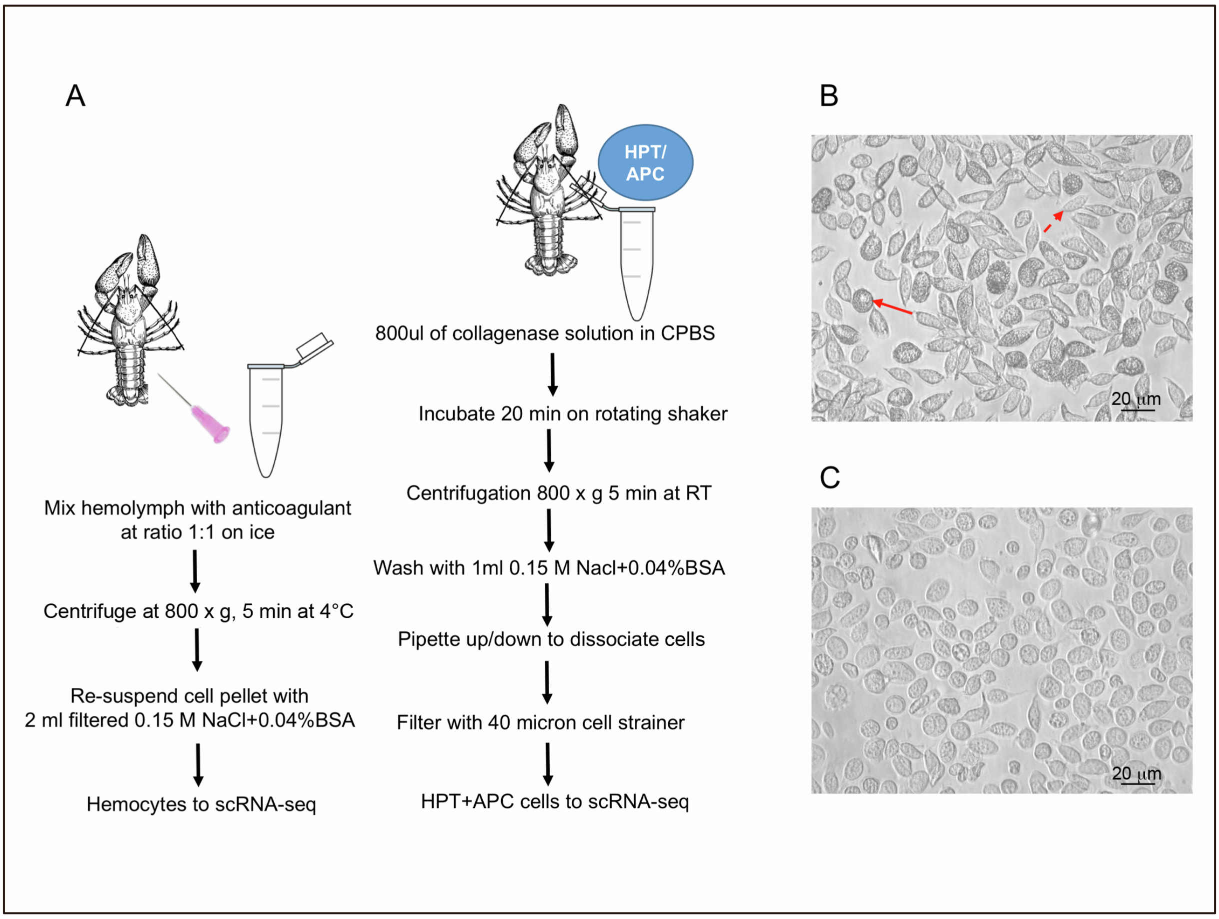

Figure S1. Cell preparation and control for single-cell RNA-sequencing.
(A) Preparation of hemocyte (left) and HPT+APC (right) samples for scRNAseq. (B) Hemocyte and (C) HPT+APC cell preparations used for single cell sequencing. Red arrow indicates a typical granular hemocyte and dashed arrow a typical semigranular hemocyte. Related to Figure 1a.

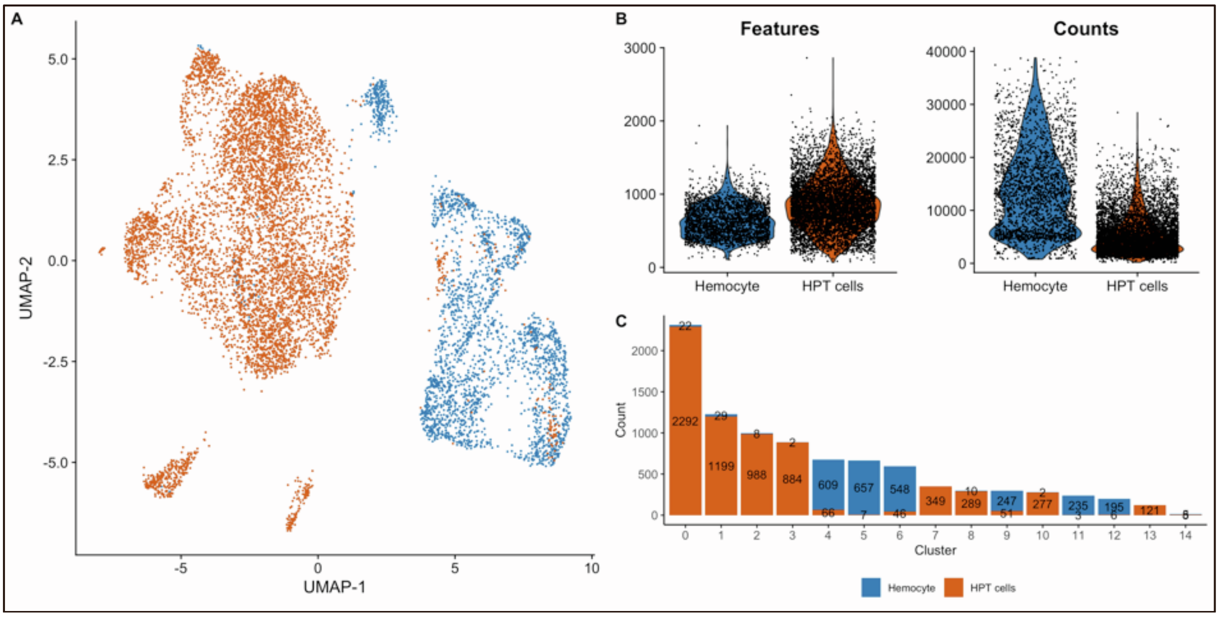

Figure S2. Clustering of different cell types in *P. leniusculus* circulation and HPT, respectively, identified by single-cell RNA-sequencing.
(A) UMAP showing the distribution of cells from each sample, HPT cells (red) and hemocytes (blue). (B) Number of features detected per cell (left) and number of counts (right) in HPT cells (red) and hemocytes (blue) respectively. (C) Number of cells in each sample and their sample origin (HPT = red, hemocytes = blue). Related to Figure 1b-d.

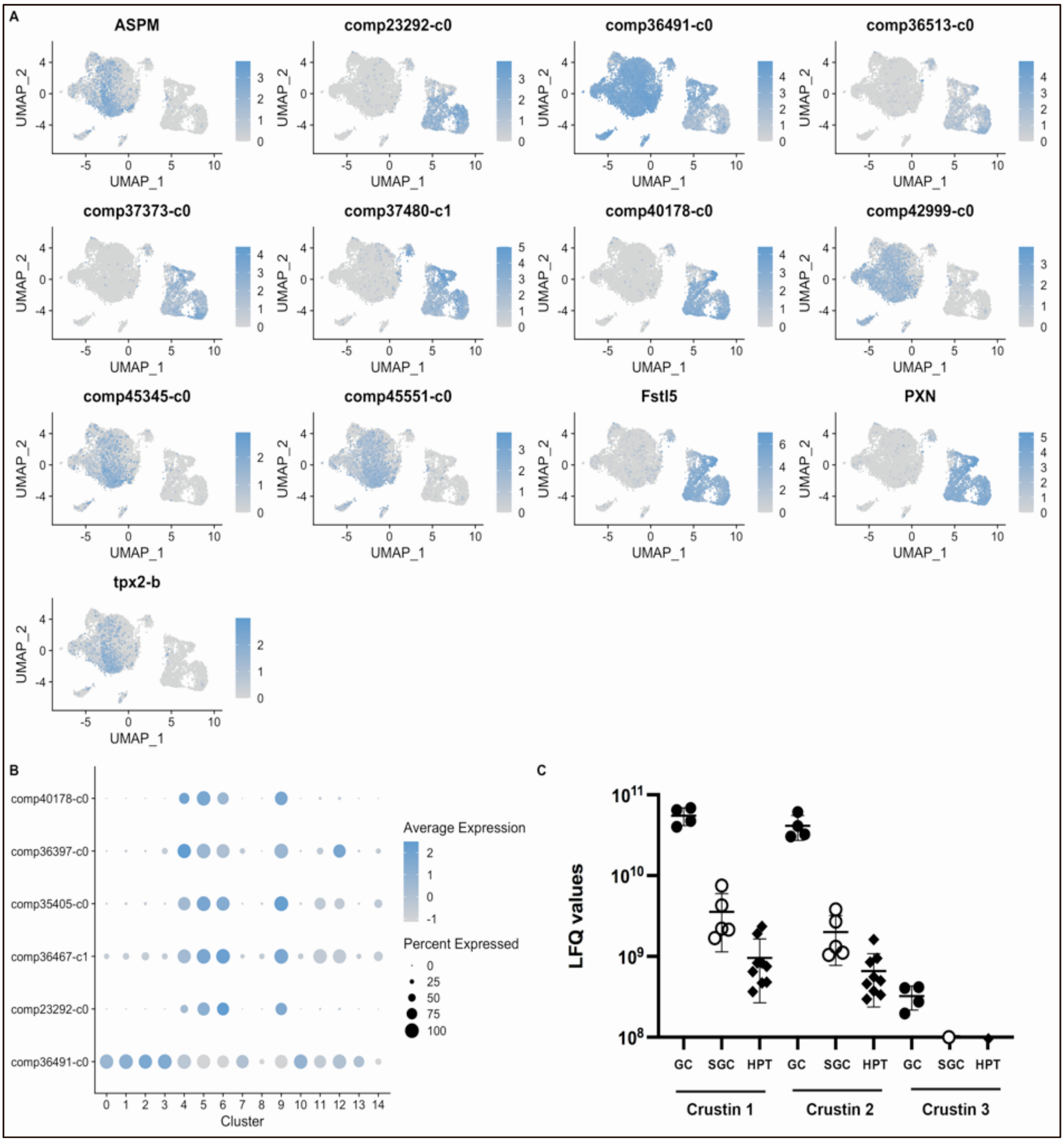

Figure S3. Major differences between hemocyte and HPT cell types.
A) UMAPs showing expression levels and cluster distribution for five selected markers for HPT cells, and eight selected markers for hemocytes. (B) Dot plots showing expression levels and cluster distribution for six crustin transcripts (some of these are in addition included in fig. 2. (C) Protein levels (LFQ= label-free quantitation) of crustin 1, crustin 2 and crustin 3 in HPT cells (n=9), semigranular cells (SGC, n= 5) and granular cells (GC, n=4) respectively. The graph is prepared in GraphPad Prism 9. Related to Figure 2.

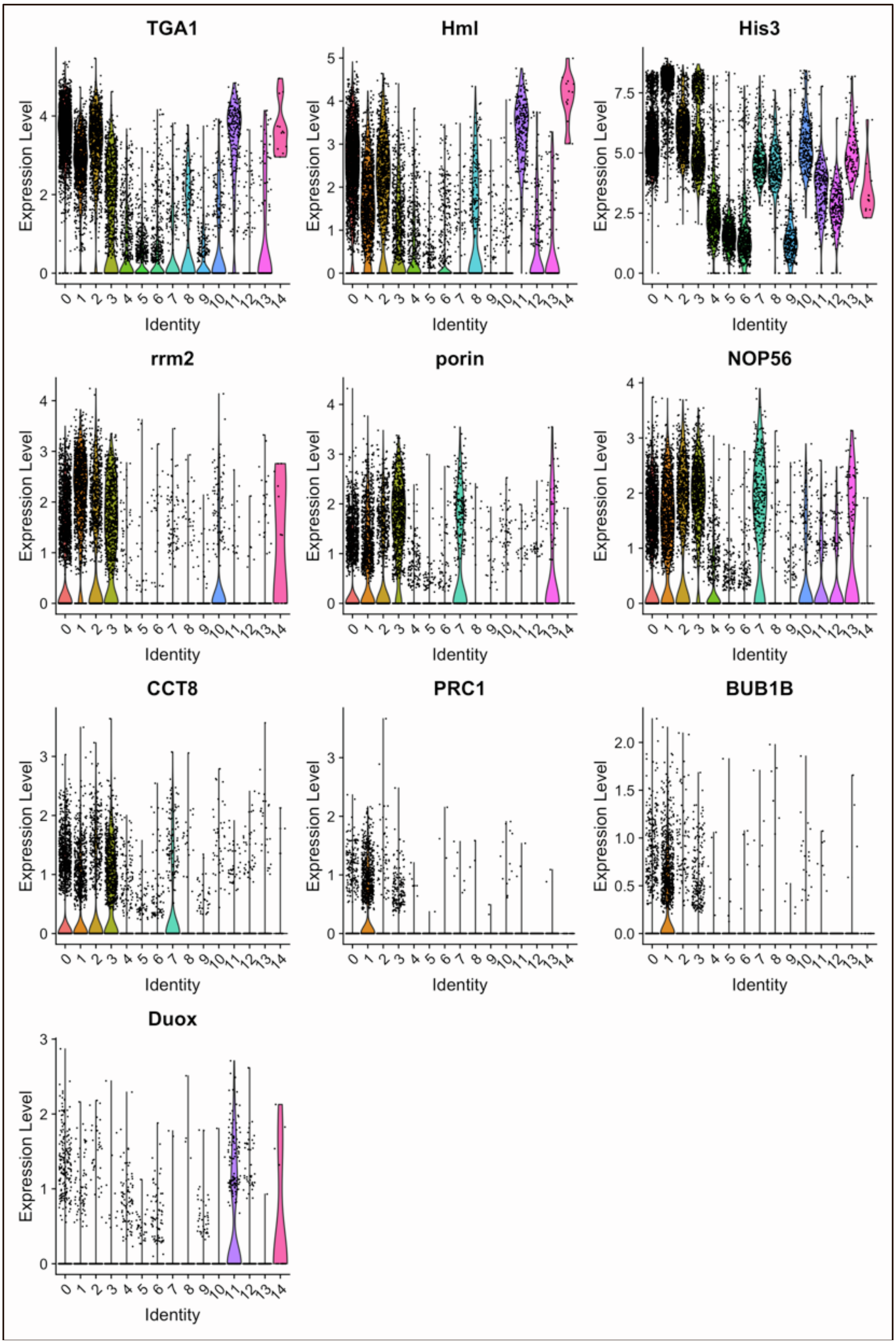

Figure S4. Expression levels and cluster distribution for selective HPT specific transcripts. Violin plots showing expression levels and cluster distribution for selective transcripts representative of HPT cells (rrm2, porin, CTT8, PRC1, BUB1B), and in HPT cells together with some hemocyte clusters (TGA1, Hml, Nop56, His3 and Duox). Related to Figure 4.

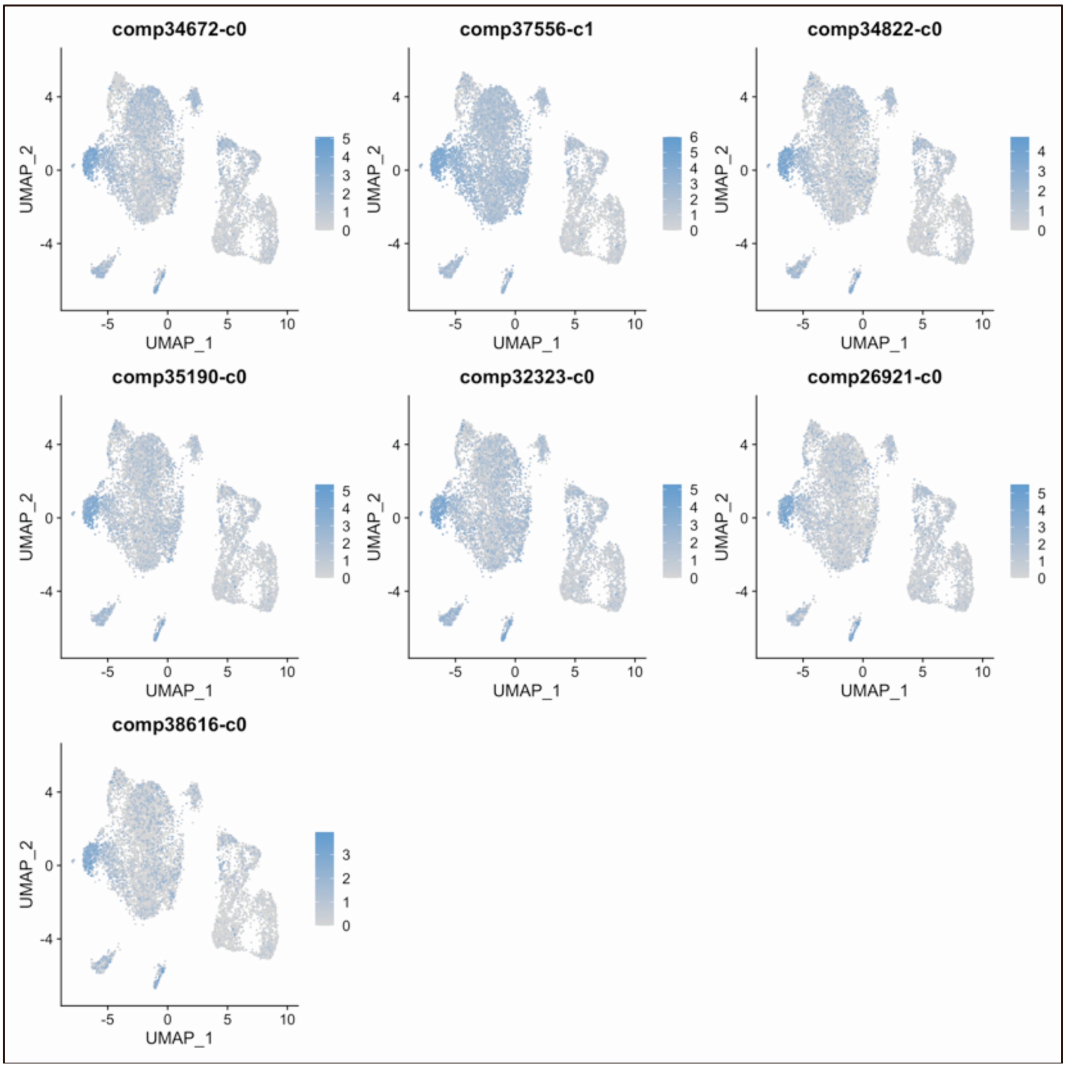

Figure S5. UMAPs showing expression levels and cluster distribution for seven transcripts of unknown function identified to be accumulated in CL7. Related to Figure 5.

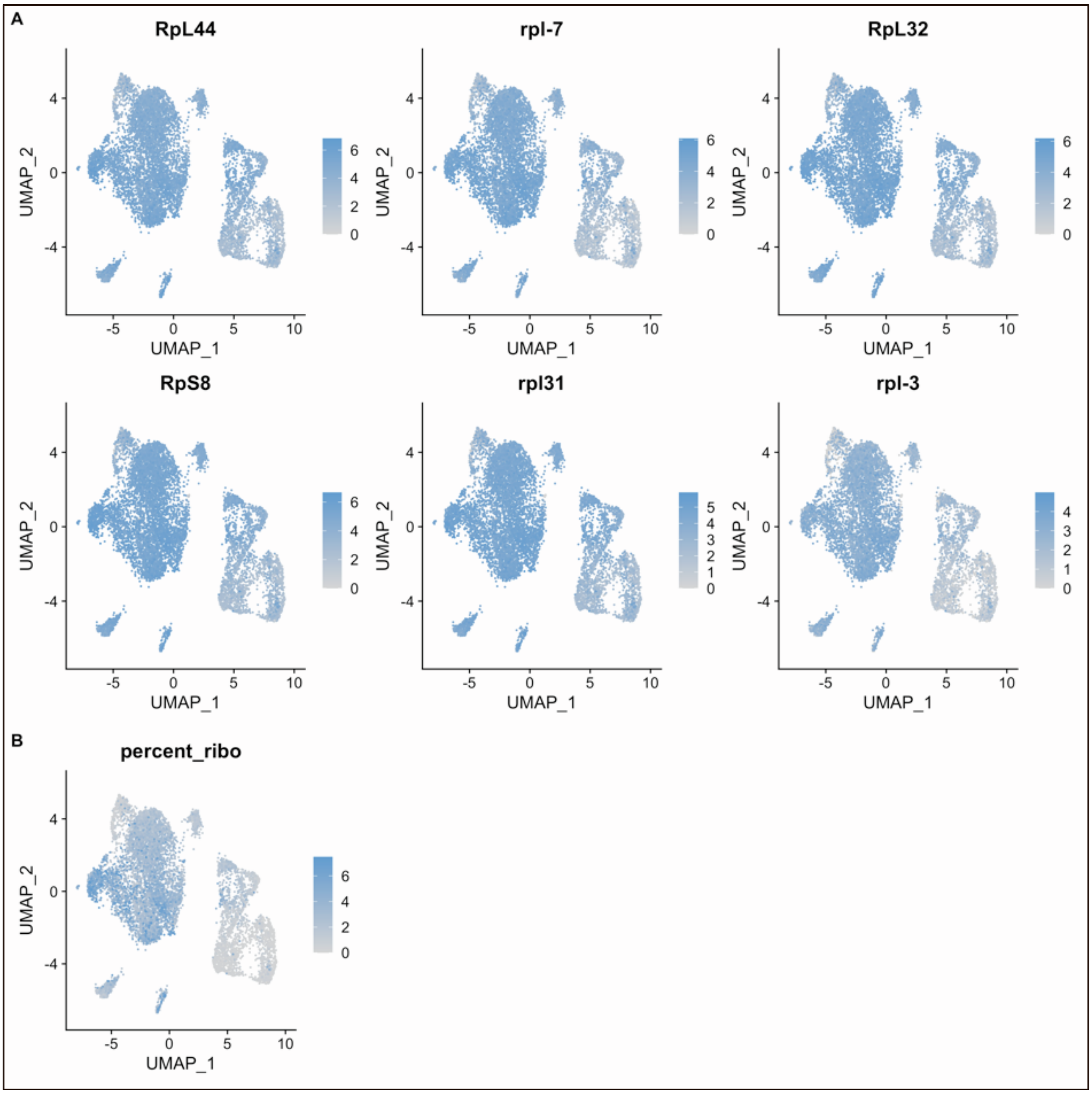

Figure S6. Distribution of transcripts encoding ribosomal proteins.
(A) UMAPs showing expression levels and cluster distribution of some selected transcripts encoding ribosomal proteins. (B) The percentage of ribosomal transcripts cluster distribution showing low percentage in mature hemocytes and in CL8. Related to Figure 1c.
